# Integrating sequence composition information into microbial diversity analyses with k-mer frequency counting

**DOI:** 10.1128/msystems.01550-24

**Published:** 2025-02-20

**Authors:** Nicholas A. Bokulich

**Affiliations:** 1 Department of Health Sciences and Technology, ETH Zurich, Zürich, Switzerland; Drexel University, Philadelphia, Pennsylvania, USA

**Keywords:** microbiome, alpha diversity, beta diversity, marker-gene sequencing, supervised learning

## Abstract

k-mer frequency information in biological sequences is used for a wide range of applications, including taxonomy classification, sequence similarity estimation, and supervised learning. However, in spite of its widespread utility, k-mer counting has been largely neglected for diversity estimation. This work examines the application of k-mer counting for alpha and beta diversity as well as supervised classification from microbiome marker-gene sequencing data sets (16S rRNA gene and full-length fungal internal transcribed spacer [ITS] sequences). Results demonstrate a close correspondence with phylogenetically aware diversity metrics, and advantages for using k-mer-based metrics for measuring microbial biodiversity in microbiome sequencing surveys. k-mer counting appears to be a suitable and efficient strategy for feature processing prior to diversity estimation as well as supervised learning in microbiome surveys. This allows the incorporation of subsequence-level information into diversity estimation without the computational cost of pairwise sequence alignment. k-mer counting is proposed as a complementary approach for feature processing prior to diversity estimation and supervised learning analyses, enabling large-scale reference-free profiling of microbiomes in biogeography, ecology, and biomedical data. A method for k-mer counting from marker-gene sequence data is implemented in the QIIME 2 plugin q2-kmerizer (https://github.com/bokulich-lab/q2-kmerizer).

**IMPORTANCE:**

k-mers are all of the subsequences of length k that comprise a sequence. Comparing the frequency of k-mers in DNA sequences yields valuable information about the composition of these sequences and their similarity. This work demonstrates that k-mer frequencies from marker-gene sequence surveys can be used to inform diversity estimates and machine learning predictions that incorporate sequence composition information. Alpha and beta diversity estimates based on k-mer frequencies closely correspond to phylogenetically aware diversity metrics, suggesting that k-mer-based diversity estimates are useful proxy measurements especially when reliable phylogenies are not available, as is often the case for some DNA sequence targets such as for internal transcribed spacer sequences.

## INTRODUCTION

Diversity metrics are frequently used in microbiome DNA sequencing surveys to quantify the microbial diversity present in individual samples (alpha diversity) as well as the similarity between samples based on the diversity present (beta diversity), e.g., in studies of microbial ecology, biogeography, as well as in biomedical research (1, 2). Several diversity metrics have been developed that incorporate phylogenetic information into calculations as a means of weighting different features based on their genetic similarity (3, 4). These are most frequently applied in high-throughput marker-gene sequencing surveys of 16S rRNA gene sequences. However, application of these methods can be hampered by some methodological limitations, e.g., the limited phylogenetic accuracy of short sequence reads (e.g., from Illumina devices) that are frequently used for DNA sequencing but can lead to erroneous phylogenies (5).

Phylogeny-aware diversity metrics can also be challenging to use with some target domains, e.g., the internal transcribed spacer (ITS) domain, which is a non-coding region located between the small and large subunit rRNA genes, and is most frequently used for molecular classification of fungi, being considered the official barcode of life for the fungal kingdom (6). Unlike the rRNA genes, which have a long history of use as phylogenetic markers, being relatively highly conserved and universally present in all domains of cellular organisms, the non-coding ITS domain has a weak phylogenetic signal due to its hypervariability, making alignment, and phylogeny estimation from distantly related clades challenging (7, 8). This limitation impedes the use of phylogenetic diversity metrics for studying fungal community ecology with ITS sequences.

Moreover, phylogenetic reconstruction can be a computationally intensive process, even from short sequence reads (e.g., 16S rRNA gene V4 domain sequences) that yield relatively imprecise phylogenies. This creates a significant bottleneck in terms of time cost and computational resources, which can be a barrier, e.g., in resource-limited settings as well as in commercial or clinical settings when turnaround time is an important factor. The primary application of phylogenetic metrics in microbiome research, e.g., relying on short sequence reads to build imprecise phylogenies, is to weight diversity metrics based on the similarity between their constituent sequences, not to infer precise evolutionary relationships *per se*.

k-mer counting provides an alternative, promising solution for incorporating subsequence information into diversity estimates and microbiome analysis. k-mers consist of the subsequences of length k that compose a given sequence, and k-mer frequencies (as well as sketching/minimization techniques based on k-mer frequencies) have been applied in diverse areas of bioinformatics and microbiome research, e.g., for taxonomic classification (9–12); phylogeny estimation (13); (meta)genome assembly, binning, and comparison (14); and supervised learning (15, 16) in both marker-gene sequence (e.g., 16S rRNA gene) and whole metagenome sequence analysis (17–20). However, k-mer counting has been largely neglected as an approach for feature processing prior to diversity analyses with different alpha and beta diversity metrics, and k-mer counting approaches have yet to be widely adopted in microbiome research.

In this work, k-mer counting was tested as a possible solution for incorporating subsequence-level information into diversity estimates and supervised learning from DNA marker-gene sequence surveys. Results demonstrate that k-mers provide meaningful information about sequence composition, enabling the incorporation of this information into diversity metrics without the high computational cost of phylogeny estimation. This suggests that k-mer-based metrics can be used alongside classical metrics as well as phylogenetically aware metrics for the quantification of diversity in complex microbial communities.

## MATERIALS AND METHODS

### k-mer counting

k-mer counting is performed from an input series of sequences (in FASTA format) using the CountVectorizer or optionally TfidfVectorizer methods implemented in scikit-learn (21). First, k-mers are counted across all sequence variants observed in a given set of samples; in this sense, each sequence is a “document” and individual tokens (k-mers) are generated as character n-grams from each individual sequence. Matrix multiplication with numpy (22) is then used to multiply the observed frequency of input sequences by the counts of their k-mer constituents to yield a feature table of k-mer frequencies per sample, enabling comparison between samples. When the TfidfVectorizer method is used, k-mers are weighted by term frequency-inverse document-frequency (TF-IDF) (23) as a method to upweight k-mer signatures that are more unique while down-weighting k-mers that are common across sequences and hence have little predictive value. The resulting method is implemented in the QIIME 2 (24) plugin q2-kmerizer (https://github.com/bokulich-lab/q2-kmerizer).

### Benchmarking data and data preparation

The Earth Microbiome Project (EMP) (1) data set was downloaded from the EMP FTP site. The “emp_deblur_150bp.subset_2k” data set (.biom md5sum = a135e5d53229bf68cb3921fd29b87531) was used, consisting of 16S rRNA gene V4 domain sequences that have been denoised to obtain unique amplicon sequence variants (ASV). ASVs observed fewer than 10 times were filtered to remove low-abundance and possibly spurious features. To obtain a reasonably sized test data set, all samples were evenly rarefied to 5,000 sequences per sample using the QIIME 2 plugin q2-feature-table (24). This yielded a total of 975 samples that passed these criteria. k-mer frequencies were counted from rarefied feature tables using both CountVectorizer and TfidfVectorizer with min_df = 10 to ignore k-mers that were observed fewer than 10 times (matching the filtering criteria used for ASVs) and (unless otherwise noted for specific tests) using max_features = 5,000 to include only the 5,000 most frequent k-mers to match the feature count of ASVs when performing side-by-side comparisons. It should be noted, however, that this is a stringent setting used for comparison purposes, and in real use cases a larger k-mer feature space should be explored.

The Global Soil Mycobiome Consortium (GSM) data (2) were downloaded from the PlutoF data repository (https://doi.org/10.15156/BIO/2263453), consisting of full-length fungal ITS sequences obtained using PacBio sequencing and pre-clustered into operational taxonomic units(OTUs) (however, these are referred to as ASVs in the text for simplicity and consistency). Samples were filtered to remove any with suspected mold or other contamination as indicated in the study metadata. RESCRIPt (25) was used to evaluate sequence length distribution and remove outliers (<300 nt and >800 nt long). To obtain a representative subsample, 100 samples were randomly sampled from each of the seven most abundant biomes represented in the data set (i.e., those with >100 samples to exclude undersampled biomes) to yield a test set consisting of 678 samples out of a total of 3,200 samples (678 < 700 because some of the representative samples were subsequently filtered due to the filtering criteria). Abundance filtering, rarifying to 3,000 sequences per sample, and k-mer counting were performed as described for the EMP data set. For the GSM data set, max_features = 10,000 was used to capture the larger k-mer space of the full-length ITS amplicons used in this study.

For plotting prevalence vs abundance curves, ASV and k-mer abundance were calculated as the total number of observations in each data set (after rarefying). Prevalence was calculated as the fraction of samples in which that feature was observed.

All k-mer counting experiments used n-gram sizes of *k* = 16 unless otherwise specified. Benchmarking the effect of k-mer size on ordination was done to select a suitable size before proceeding with further analysis. The selection of *k* = 16 as a suitable default was done based on a visual assessment of principal coordinate analysis (PCoA) cluster quality and the use of silhouette scores (see below) as qualitative assessments of cluster separation. All statistical analyses and comparisons across metrics as described below were only performed after the selection of a k-mer length to use for all experiments. This process was followed to avoid “p-hacking” by selecting a k-mer size that gives the best statistical result in a given experiment.

### Diversity analysis

Alpha diversity (observed features, Shannon entropy, Faith’s Phylogenetic Diversity) and beta diversity (Bray Curtis dissimilarity, Jaccard distance, Aitchison distance, un/weighted UniFrac [3]) were calculated using QIIME 2 version 2024.5 with the q2-diversity plugin (24). Non-phylogenetic metrics (observed features, Shannon, Bray Curtis, Jaccard, Aitchison) were calculated on rarefied feature tables or k-mer frequency tables. Phylogenetic metrics were calculated only on ASV frequencies in the EMP data set, using the pre-computed phylogeny provided by the EMP (file “emp150.5000_1000_rxbl_placement_pruned75.tog.tre,” md5sum = 6cbed1b475d242bf388ef13d3f0fe12c). Permutational multivariate analysis of variance (PERMANOVA) tests (26) were performed using the q2-diversity plugin to test for significant differences in beta diversity between EMP ontology level 3 (EMPO 3) categories, which categorize samples based on general sample types. ANOVA tests with false discovery rate correction for multiple tests, as implemented in q2-longitudinal (27), were used to test for significant differences in alpha diversity.

Mantel tests were performed to test the correlation between pairwise distances calculated by each distance metric and feature processing method; Spearman correlation was used to correlate these distances and a higher Rho value indicates a closer correlation. Alpha diversity correlations between metrics and methods were measured using Pearson correlation and Spearman correlation implementations in SciPy (28). Alpha correlation and beta diversity PCoA plots were generated using the seaborn package (29). To test the goodness of fit between PCoA coordinates calculated for each metric and feature processing method (k-mers vs ASVs), Procrustes analysis (30) was performed using the q2-diversity plugin. Procrustes analysis uses translation, rotation, and uniform scaling to find the best fit between two objects (in this case PCoA coordinates) that minimizes the sum of squares of pointwise differences (the M^2^ statistic reported; lower indicates a better fit). In some parameter testing experiments, silhouette coefficients (31) were calculated as a measure of cluster tightness and separation, using the silhouette_samples function in scikit-learn.

### Supervised learning

Random forest classifiers (32) with 500 trees were trained using 10-fold nested cross-validation using the QIIME 2 plugin q2-sample-classifier (33). Models were trained on rarefied ASV or k-mer frequency tables and tested on a hold-out set at each fold. Accuracy was measured at each fold by comparing true vs predicted values. Receiver operating characteristic (ROC) curves were calculated for each model using the predicted class probabilities and used to measure the area under the curve (AUC). K-nearest neighbors models (*k* = 3, 10-fold cross-validation) were used to test predictive accuracy for sample classification based on pairwise UniFrac distances. Although this is a different model and approach, it allows a comparison of predictive accuracy between ASV or k-mer frequencies vs UniFrac diversity estimates for predicting sample metadata categories.

### Computational performance

Runtimes were measured using a single core on a Macbook Pro with a 2 GHz Quad-Core Intel Core i5 processor and 16 GB RAM. The EMP and GSM data sets were used to provide test data from 150 nt long 16S rRNA gene V4 sequences and ~800 nt long PacBio sequences, respectively. Sub-samples of 100, 500, 1,000, and 10,000 sequences were taken from each data set to test performance with different-sized queries. k-mer counting (*k* = 7) was performed as described above with TF-IDF and 5,000 maximum features. The “align_to_tree_mafft_fasttree” action in the QIIME 2 plugin q2-phylogeny was used to perform pairwise alignment with mafft (34) followed by phylogeny estimation with fasttree2 (35).

## RESULTS

The EMP data set was selected for testing here as a significant global data set representing most key sample categories, consistently categorized using the EMP ontology, EMPO. This data set consists of 16S rRNA gene V4 domain ASVs.

### k-mer diversity corresponds to phylogenetically aware diversity estimates

**Fig 1:**
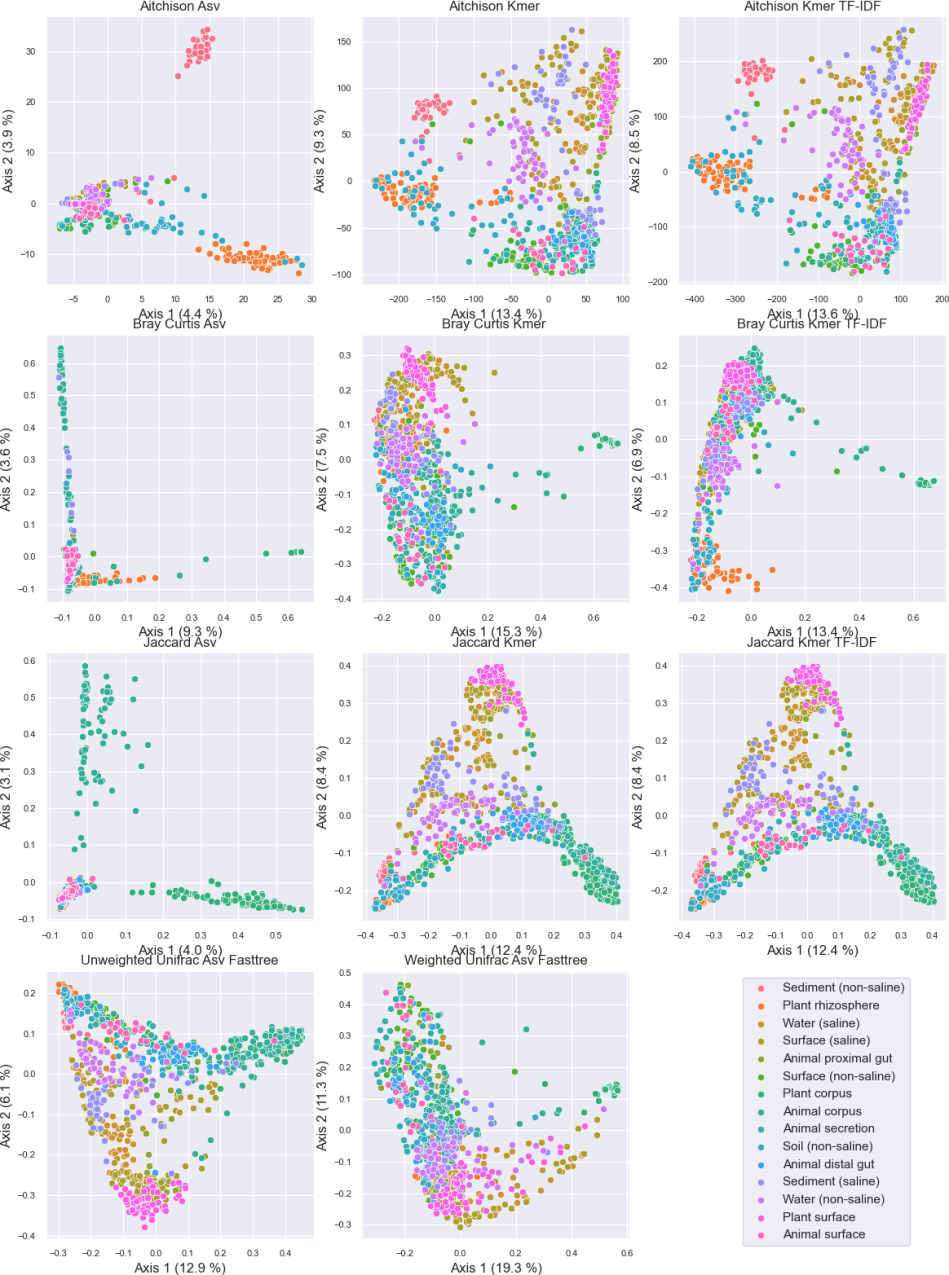
Principal coordinate analysis comparison of sample compositions based on different beta diversity metrics and features (ASVs vs 16-mers). Samples are colored by EMPO 3 category. Axis labels indicate the percent variance explained. Note that Jaccard distances are identical for k-mer profiles with and without TF-IDF, as TF-IDF was used to weight k-mers based on importance, but not for feature selection. Hence, both profiles contain the same k-mers so binary metrics (e.g., Jaccard) yield identical distances, but weighted metrics (e.g., Bray Curtis) vary due to the TF-IDF weightings.

**Fig 2:**
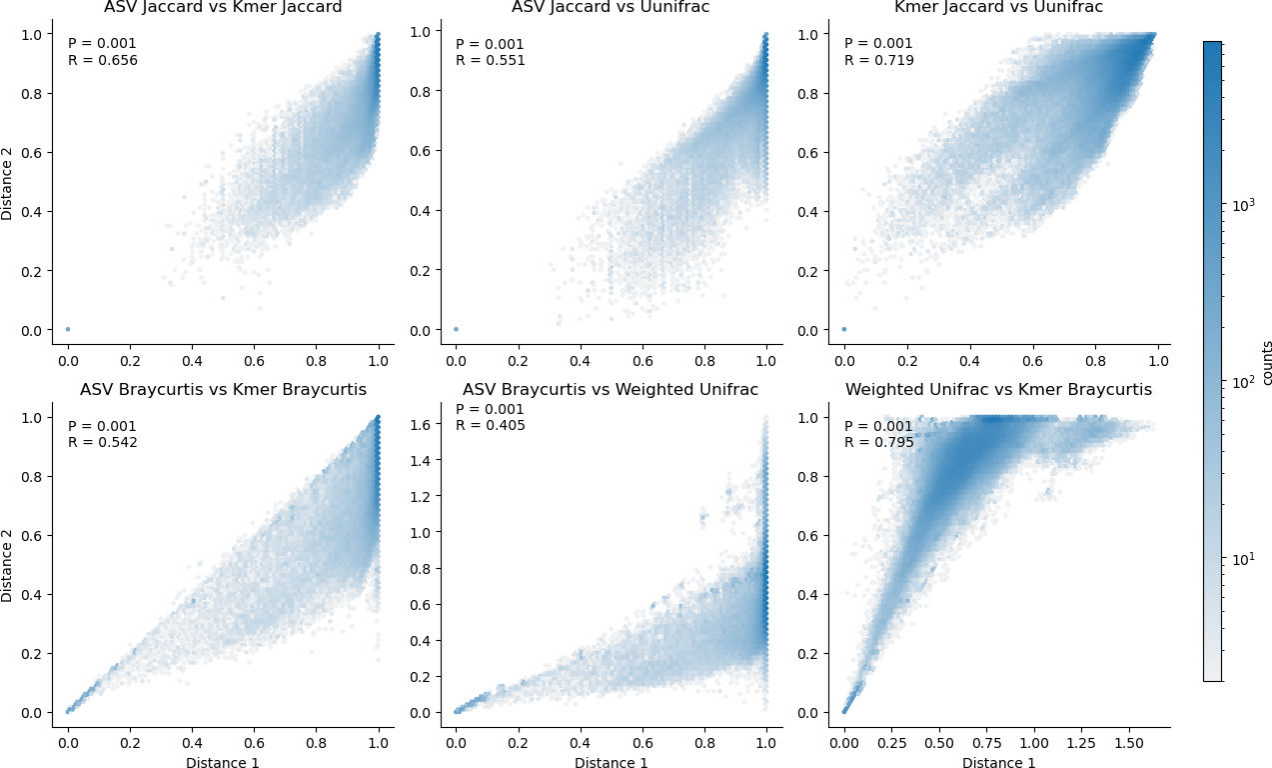
k-mer-based diversity metrics correlate with phylogeny-aware metrics. Distance-distance correlation plots between selected conventional, phylogeny-aware, and k-mer-based (*k* = 16) beta diversity metrics demonstrate that k-mer-based metrics correlate more closely with phylogeny-aware metrics than conventional metrics do. Mantel test results (Spearman *R* and *P*-values) for each comparison are shown in each panel.

k-mer counting yields beta diversity distances that correlate well with those derived from the phylogenetically aware beta diversity metric UniFrac (Fig. 1 and 2; Table S1; Mantel correlation with weighted UniFrac Spearman *R* = 0.795). Mantel tests indicate that this correlation is stronger than the correspondence between k-mer frequency-based and ASV-based distances using non-phylogenetic diversity metrics (Spearman *R* = 0.542), as well as between ASV-based and Unifrac distances, which only show weak correspondence (Spearman *R* = 0.405). Ordinations based on k-mer frequencies are qualitatively more differentiable between sample types in the EMP data set (Fig. 1), and yield larger PERMANOVA *R*^2^ values for differentiating EMPO 3 categories, indicating better quantitative differentiation between sample types than for ordinations based on ASV frequencies when using the same metrics (Table S2 and S3). The k-mer frequency-based PERMANOVA *R*^2^ values for unweighted (Jaccard) and weighted (Bray Curtis) diversity metrics are also in close correspondence with those for unweighted and weighted UniFrac, respectively (Table S2), indicating that k-mer frequencies and phylogeny-aware metrics have a similar degree of efficacy for differentiating communities based on subsequence-level information, as can be expected from the closely correlated ordinations (Table S1). Both UniFrac and k-mer-based distance metrics consistently yield higher PERMANOVA *R*^2^ values (i.e., higher percent variation explained by EMPO 3 class; mean *R*^2^ differences of 0.073–0.110) than conventional diversity metrics based on ASV frequencies (Table S3). UniFrac metrics demonstrated slightly higher mean *R*^2^ values than k-mer-based metrics (mean *R*^2^ = 0.025–0.036) (Table S3).

Supervised learning was also used to determine the predictive value offered by different feature processing methods (ASVs vs k-mers generated from those ASVs) as well as vs UniFrac distance estimates from ASVs. Random Forest classification of EMPO 3 categories (using 10-fold nested cross-validation with 500 trees at each fold) demonstrated that predictive accuracy was marginally improved when models were trained on k-mers either with (accuracy = 0.854) or without TF-IDF (accuracy = 0.855) compared to ASVs (accuracy = 0.828), though ROC AUC was equivalent for the three approaches (micro-averaged AUC = 0.99). This exceeded the accuracy for k-nearest neighbors classification of EMPO 3 categories based on weighted UniFrac (accuracy = 0.787) and unweighted UniFrac (accuracy = 0.838). Hence, the expanded feature space offered by k-mer frequencies may increase classification accuracy in some contexts; further optimization (e.g., through feature selection) could be done to further improve performance, but will be data set dependent and is out of scope in this work. In any given experiment, ASVs and k-mer frequencies both are recommended as inputs for supervised learning models to determine the predictive power offered in the context of that experiment.

### k-mer length and other parameters influence diversity estimates

**Fig 3:**
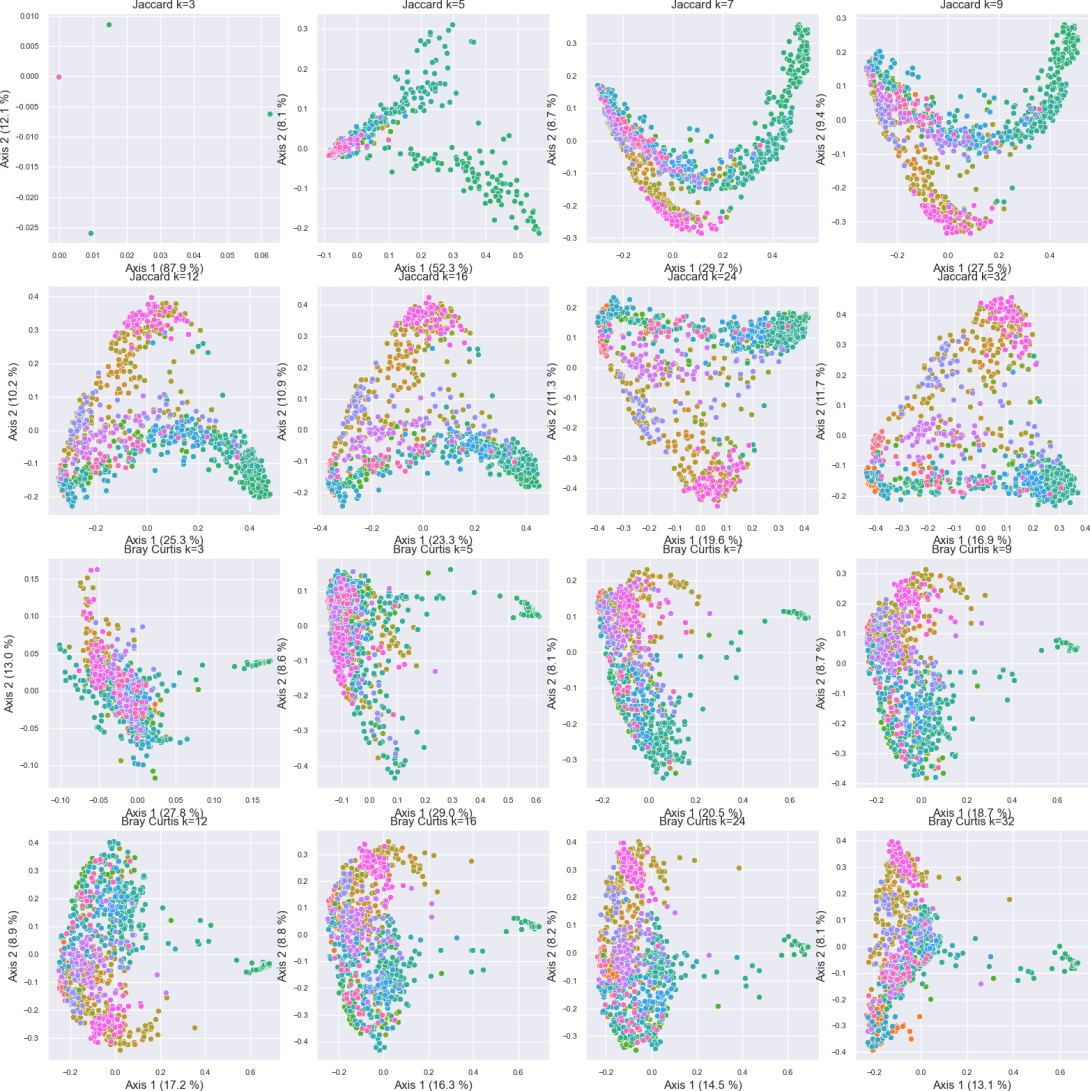
Influence of k-mer length on PCoA ordinations based on Jaccard distance (top two rows) and Bray Curtis dissimilarity (bottom two rows). Samples are colored by EMPO 3 category (see legend in Fig. 1). Axis labels indicate percent variance explained.

The impact of k-mer length (i.e., n-gram size) on beta diversity ordinations (Fig. 3), Silhouette scores (Fig. S1), and Procrustes goodness of fit (Table S4) with phylogenetically aware beta diversity metrics was tested on the same data set, comparing weighted (k-mer Bray Curtis vs ASV weighted UniFrac) and unweighted (k-mer Jaccard vs ASV unweighted UniFrac). For this test, TF-IDF vectorization was performed while constraining the feature table to the top 10,000 most important k-mers so that the k-mer count is kept constant across all tests. Results indicate that k-mer lengths between *k* = 12 and *k* = 32 yielded similar Jaccard distance ordinations (Fig. 3) to UniFrac distance ordinations (Fig. 1), with good Procrustes fits at values of *k* ≥ 7 (Table S4). Mean Silhouette coefficients (31), a measure of cluster separation and density, were used to measure the separation of clusters based on EMPO 3 categories in this experiment. Silhouette scores significantly increased until *k* = 7, and continued to slightly increase until *k* = 16 (Fig. S1) hence *k* = 16 was kept for all other tests in this work. Nevertheless, the influence of k-mer length on diversity estimates may vary by target domain and depend on the underlying diversity, and hence in any given experimental scenario it is recommended to repeat this process when applying this method in different experimental contexts. However, very small k-mer lengths reduce the feature space overall, leading to a small number of highly prevalent features, resulting in poorer diversity estimates and limited ability to differentiate samples; very large k-mers may be expected to have the same effect. Other small benchmarks were performed to examine how robust k-mer counting is to sampling depth and filtering constraints, and are described in the supplemental material (Fig. S3 to S5).

### k-mer diversity correlates with ASV-based alpha diversity estimates

**Fig 4:**
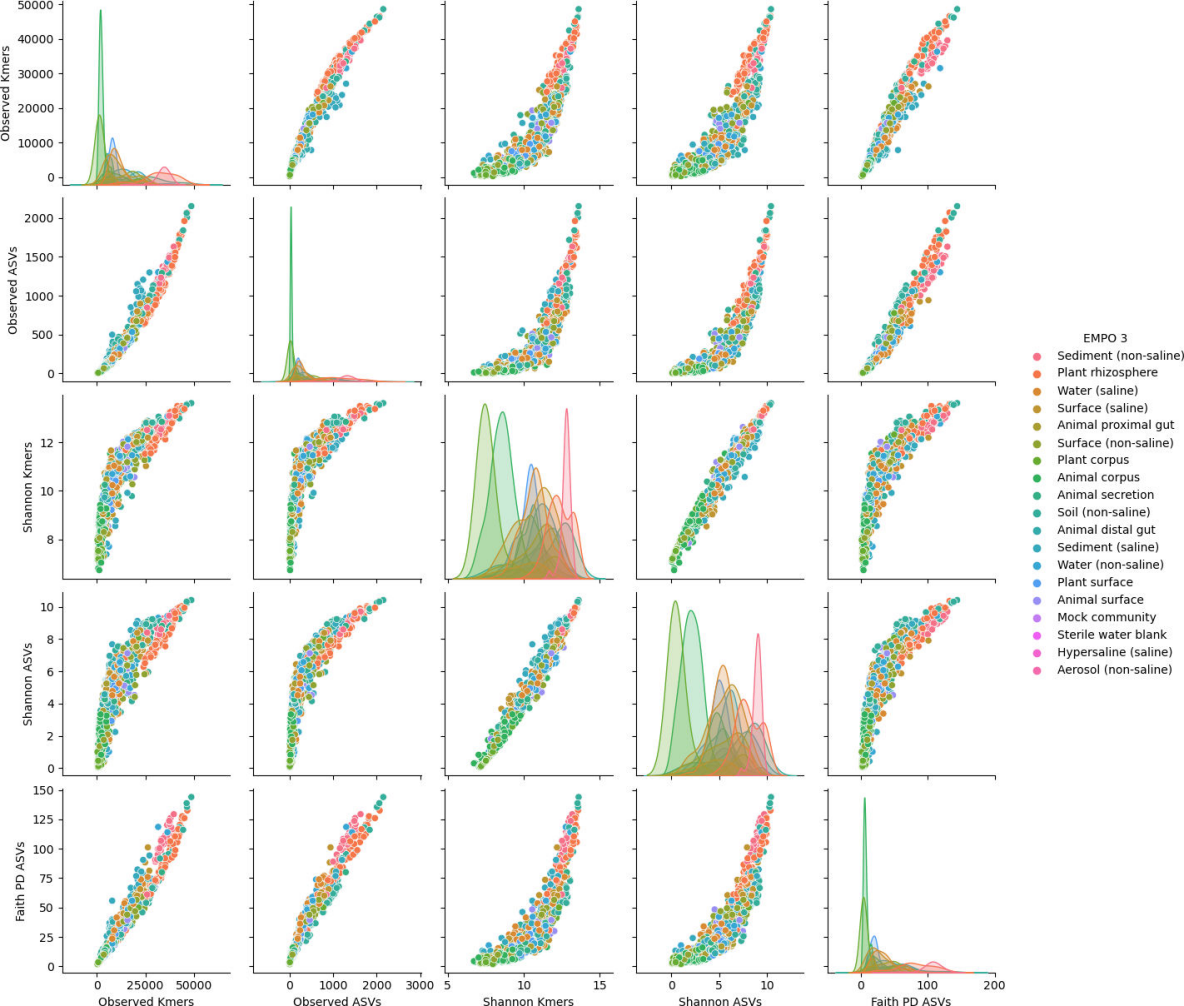
Pairplot of correlations between k-mer- and ASV-based alpha diversity estimates in the Earth Microbiome Project data set. k-mers were generated with an n-gram size of *k* = 16.

Next, alpha diversity estimates for the EMP data were compared between 16-mers (observed k-mers, Shannon entropy) and ASVs (observed ASVs, Shannon entropy, and the phylogenetically aware metrics Faith’s phylogenetic diversity). Overall, good correlations were seen between all metrics, indicating that richness, entropy, and phylogenetic diversity are correlated within microbial communities on a global scale (i.e., within the EMP data set) and that k-mer diversity is closely correlated to ASV diversity (Fig. 4; Table S5). A close correlation was observed between observed k-mers and Faith’s phylogenetic diversity (Spearman *R* = 0.986, *P* < 0.001), though close correlations were observed between all metrics, regardless of feature type (Table S5).

Hence, k-mer diversity could be used in addition or instead of phylogeny-based diversity metrics as a measurement of subsequence diversity present within a community. This comes with the added advantage that k-mer-based metrics can be directly compared between data sets when parameters (e.g., rarefaction depth and k-mer length) are kept consistent, whereas phylogenetically aware metrics are only comparable when an identical phylogeny was used, which can limit comparability of results in the literature. However, this comes at the cost of interpretability; whereas richness measurements are easily interpretable (e.g., number of unique species or sequence variants observed in a sample), k-mer richness does not map to such an intuitive value, though arguably this is still more intuitive and interpretable than for some phylogenetic alpha diversity metrics, e.g., which correspond to the cumulative amount of branch length on a tree (depending on the phylogeny and the unit of measurement).

A word of caution regarding these results: researchers should be particularly careful about applying k-mer counting prior to alpha diversity assessments, and interpreting these results. Whereas richness and Shannon are classically used to assess the richness and diversity/evenness of species or sequence variants, applying these metrics to k-mers requires a distinct interpretation; these results do not infer the actual number and evenness of species or sequence variants, but the number and evenness of k-mer variants observed, which is then related to the amount of genetic diversity present in a community. Hence, such metrics provide useful information for relativistic interpretations (e.g., community A has higher k-mer diversity than community B), but the absolute values measured cannot be used to infer the number of species present. Transforming an OTU or ASV into k-mer frequencies will yield multiple counts from a single sequence, and sequences with higher k-mer diversity will yield a higher number of unique k-mers (though not a higher total count if the sequences are of a fixed length); hence, k-mer counting is only meaningful when comparing sequences derived from the same genetic target, and could lead to misleading results if, e.g., k-mer richness is inflated by the presence of a small number of highly variable or noisy sequences. Moreover, feature selection approaches (e.g., TF-IDF or aggressive prevalence-based filtering) should be avoided, as any alpha diversity assessments are then based on an arbitrary selection of the most “important” (e.g., most frequent or most characteristic) k-mers instead of the full set. Finally, as some diversity metrics pose strict assumptions about the underlying features, k-mer counts may not be suitable for some metrics. The assumptions of any metric and statistical test should always be checked to validate compatibility.

### k-mer diversity incorporates subsequence information into fungal ITS diversity estimates

To evaluate the utility of k-mer counting in fungal ITS studies, the GSM data set was selected as a test data set, consisting of PacBio full-length fungal ITS sequences from soil microbiomes collected from different locations, and categorized according to biome.

**Fig 5:**
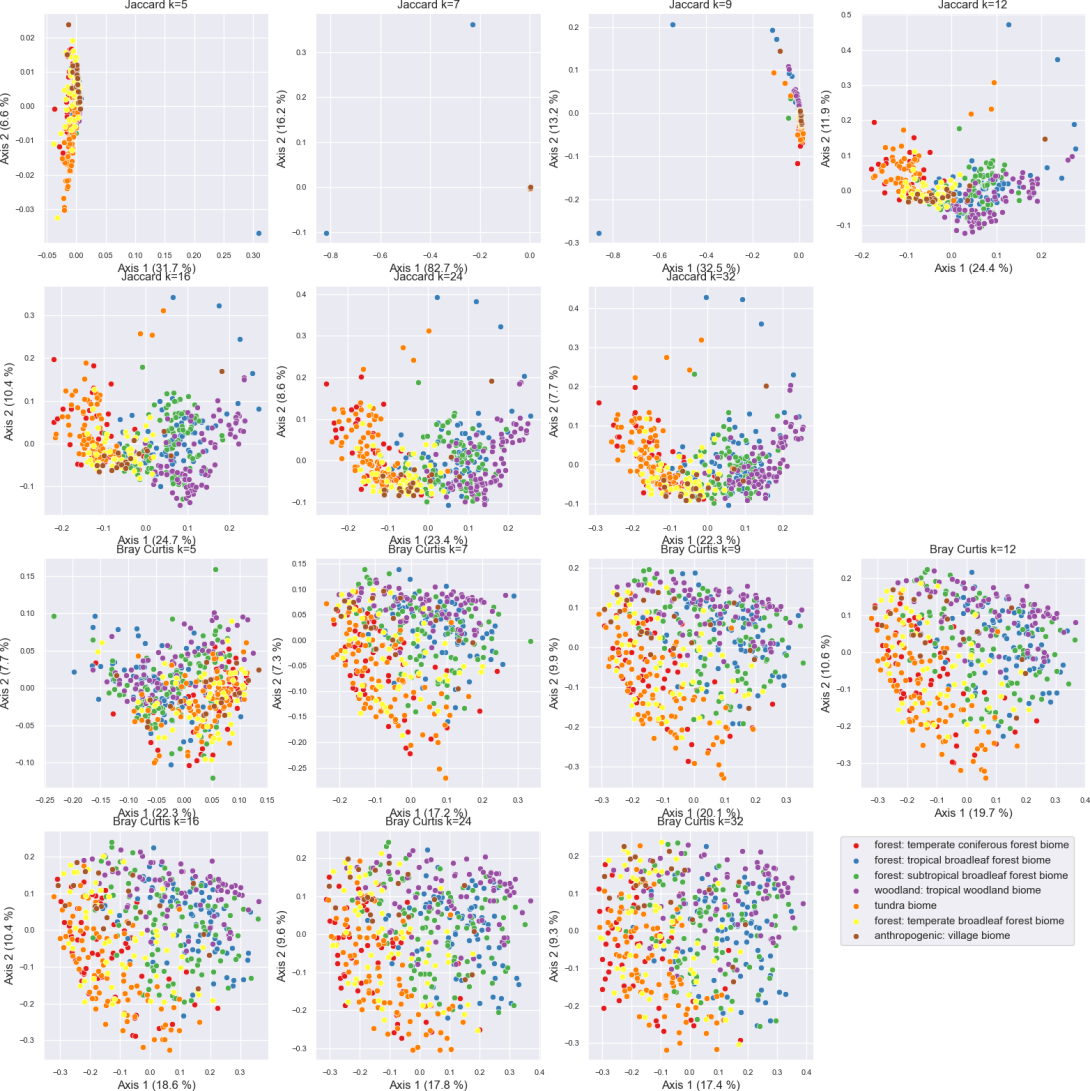
Influence of k-mer length on PCoA ordinations based on Jaccard distance (top two rows) and Bray Curtis dissimilarity (bottom two rows) in the Global Soil Mycobiome data set. Samples are colored by biome category. Axis labels indicate the percent variance explained.

Different k-mer lengths were tested to determine how this impacted weighted (Bray Curtis dissimilarity) and unweighted (Jaccard distance) beta diversity in the GSM data set. Low k-mer lengths led to unweighted distances that were distorted with outliers and weighted metrics appeared less sensitive to k-mer length (Fig. 5). Silhouette scores, as a measure of clustering quality, demonstrated a mean increase and significantly higher scores up until *k* = 16 (Wilcoxon rank-sum *P* < 0.05) (Fig. S6). Thus, a k-mer length of 16 was chosen for further analyses with the GSM data set.

**Fig 6:**
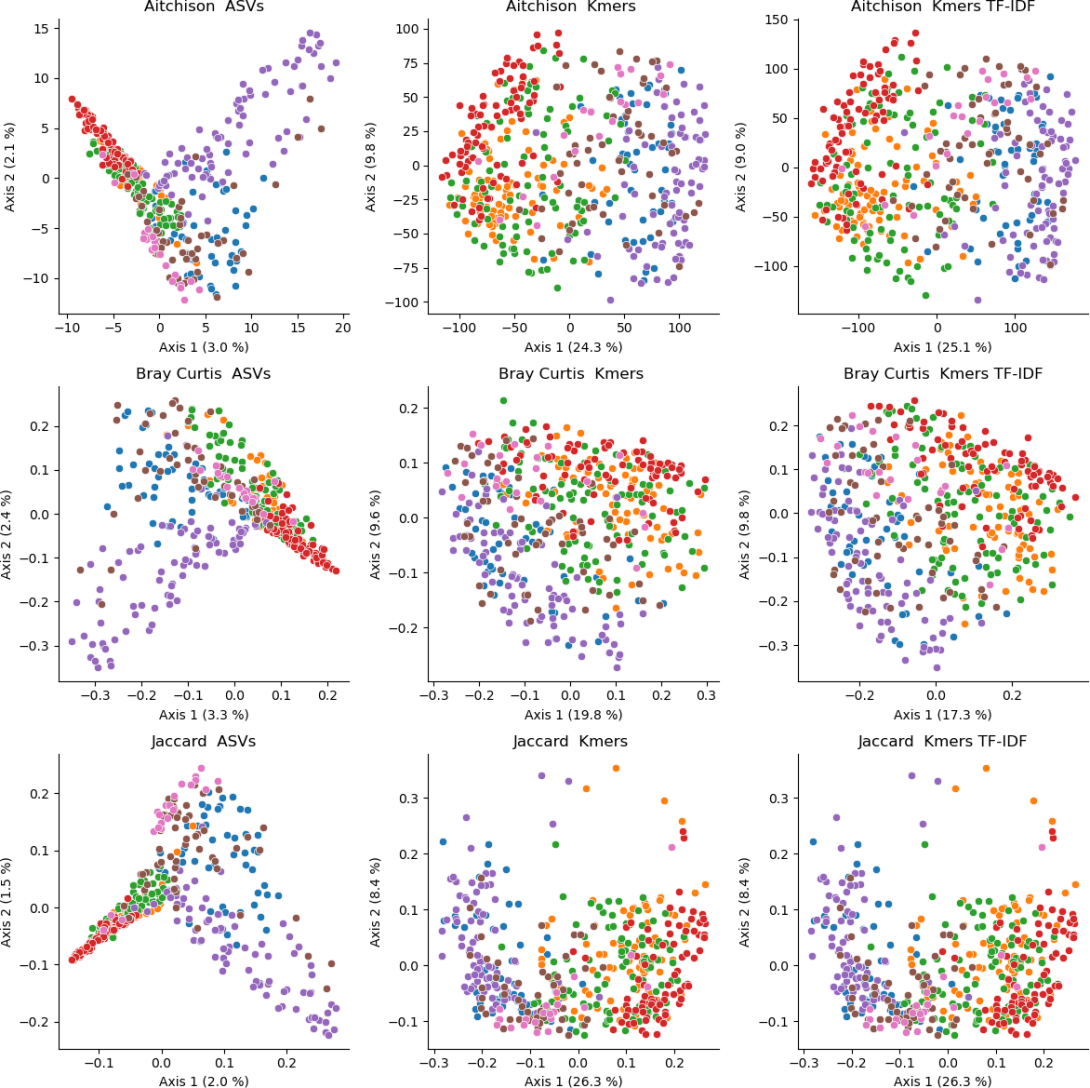
Principal coordinate analysis comparison of sample compositions based on different beta diversity metrics and features (OTUs vs k-mers) in the Global Soil Mycobiome data set. Samples are colored by biome category (see Fig. 4 for legend). Axis labels indicate the percent variance explained. Note that Jaccard distances are identical for k-mer profiles with and without TF-IDF, as TF-IDF was used to weight k-mers based on importance, but not for feature selection. Hence, both profiles contain the same k-mers so binary metrics (e.g., Jaccard) yield identical distances, but weighted metrics (e.g., Bray Curtis) vary due to the TF-IDF weightings. k-mers were generated with an n-gram size of *k* = 16.

Beta diversity was calculated as described for the EMP data set, comparing ASV-based vs k-mer-based diversity using weighted (Bray Curtis dissimilarity), unweighted (Jaccard distance), and compositionally aware metrics (Aitchison distance) (Fig. 6). The ASV-based PCoA ordinations exhibited very low percentage variance explained (maximum 5.7% on the first two PCs) and highly distorted plots with poor separation of several sample classes (i.e., biome types). On the other hand, k-mer-based PCoA ordinations all demonstrated good separation of sample types, high percentage variance explained (between 29.4% and 34.7% in the first two PCs), and smooth, undistorted ordinations. PERMANOVA tests demonstrated similarly superior performance of k-mer-based metrics. Both ASV-based and k-mer-based metrics yielded significant PERMANOVA results (*P* = 0.001; Table S6), but k-mer-based metrics consistently yielded significantly higher effect sizes (Wilcoxon rank-sum *P* < 0.001; mean *R*^2^ values between 0.067 and 0.121 higher than the corresponding effect size for ASV-based distances/dissimilarities) (Tables S6 and S7). k-mer TF-IDF filtering did not have a significant impact on results.

Hence, k-mer counting, when correctly parameterized, appears to be an effective strategy to incorporate subsequence-level information into community diversity estimates based on ITS sequences, leading to better discriminatory power than ASV (or OTU-based) methods, which do not incorporate sequence similarity into their estimates, and hence treat all features as equally unrelated entities. This limits discriminatory power, or may even artificially amplify differences between samples, e.g., when differences between samples are driven by genetically similar organisms (e.g., different strains within the same species). For this reason, phylogenetically aware metrics have been recommended for use alongside classical beta diversity metrics to evaluate how genetic diversity influences beta diversity (3). In lieu of a robust method for measuring phylogenetic diversity with ITS sequences (as a non-coding, phylogenetically uninformative region), k-mer counting provides a solution for incorporating subsequence-level information into diversity estimates to fill this niche. This is not intended as a replacement for ASV- or OTU-based diversity estimates, but as a complimentary approach, as the interpretation is distinct (i.e., distances based on shared species composition vs shared genetic subunit composition).

**Fig 7:**
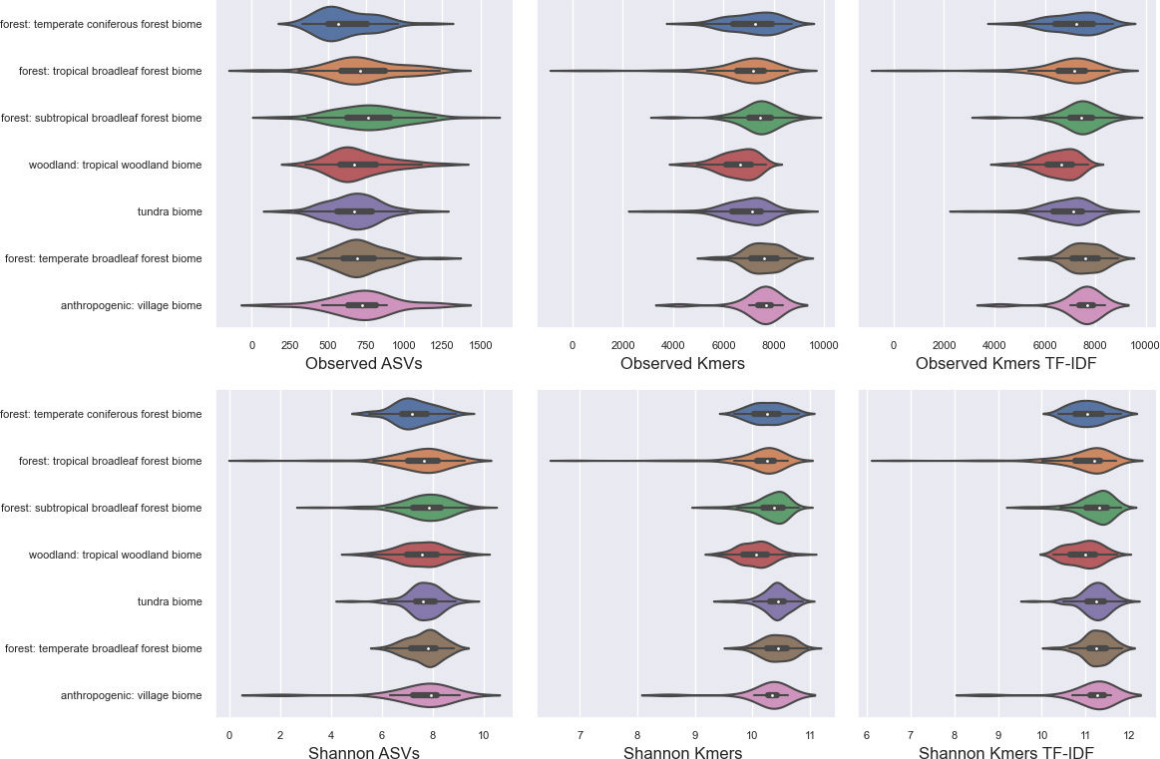
Alpha diversity estimates for the top seven biomes in the Global Soil Mycobiome data set. k-mers were generated with an n-gram size of *k* = 16.

Next, alpha diversity was measured using several metrics based on both ASVs and k-mer frequency. As ITS is a non-coding, high-entropy region, it is not informative as a marker to estimate phylogenies between highly disparate clades, and therefore it would not be meaningful to calculate phylogenetically aware diversity metrics (e.g., UniFrac or Faith’s PD) with the GSM fungal ITS data set for comparison vs k-mer-based diversity estimates as shown above for the EMP data set to demonstrate the close correspondence between these methods. Thus, with the GSM data set the next analysis was instead to examine how ASV- or k-mer-based alpha diversity estimates lead to different conclusions about community-level diversity. Results demonstrate that k-mer-based diversity estimates have lower relative variance than ASV-based diversity estimates (Fig. 7), leading to better discrimination between groups and more significant pairwise differences (Fig. S7). These correspond to results from synthetic communities that demonstrate the ability of k-mer-based metrics to detect differences in alpha and beta diversity that are driven by the presence of genetically distinct clades within a community (Supplemental Material; Fig. S8 to S10; Tables S8 and S9).

These results do not imply that k-mer-based metrics are superior to classical ASV-based metrics, or should serve as a replacement. Instead, these should be used as complementary approaches, similar to how phylogenetic alpha diversity metrics are used as complementary metrics for 16S rRNA gene diversity. The underlying features are fundamentally different and would not be expected to yield equivalent results, but instead give complementary views of the diversity. Importantly, ASV richness (observed ASVs) has the advantage of being easily interpretable. Hence, these are proposed as complementary, not competing approaches for feature processing. Moreover, k-mer counting fills the niche for incorporating subsequence-level information into fungal ITS diversity metrics (both alpha and beta), and here demonstrates how such information can lead to different interpretations vs ASV (or OTU-based) metrics.

### k-mer counting is computationally inexpensive

One motivation for k-mer counting is to provide a computationally inexpensive process for incorporating sequence information into diversity estimates. Precise phylogeny estimation (and the pairwise sequence alignment step that precedes it) can be very computationally intensive on large data sets, creating a significant resource and time bottleneck for many researchers. Runtimes were compared between k-mer counting and pairwise alignment with mafft (34) followed by phylogeny estimation with fasttree2 (35). Runtimes were calculated as a function of sequence count (100, 500, 1,000, or 10,000 sequences) and sequence length (150 nt long 16S rRNA gene V4 sequences from EMP or ~800 nt long PacBio sequences from GSM).

**Fig 8:**
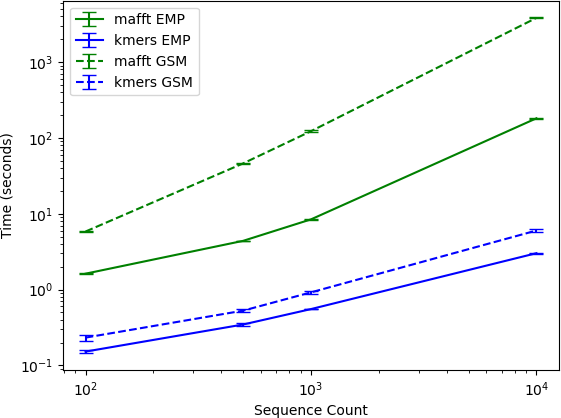
Analysis runtime (seconds) for mafft pairwise alignment + fasttree2 phylogeny estimation (green) vs k-mer counting (blue) as a function of sequence count and length. The *X* and *Y* axes are displayed in logarithmic scale.

Results show that k-mer counting is computationally inexpensive, scaling linearly and taking seconds to process 10,000 sequences and their frequencies in hundreds of samples from the EMP and GSM data sets, compared to many minutes to over an hour for alignment and phylogeny estimation with mafft and fasttree2 (Fig. 8). As expected, the longer GSM sequences led to significant increases in runtime for mafft + fasttree, but only a minimal increase for k-mer counting. This indicates that k-mer counting takes between 1 and 3 orders of magnitude less runtime on equivalent resources, compared to mafft + fasttree, depending on the number and length of sequences. Moreover, this is just a 10k sequence subset of the EMP and GSM data sets; the full studies reported 307,572 and 722,682 unique sequences, respectively, which would lead to significantly longer runtimes for data sets of that scale. However, all tests were performed with a single core for the purposes of benchmarking, and so in practice, all runtimes could be reduced via parallelization when sufficient resources are available.

## DISCUSSION

k-mer frequency counting is proposed here as an approach for sequence processing prior to beta diversity, supervised learning, and (with some important caveats) alpha diversity analyses in sequence-based microbiome profiling. This is proposed as a complementary method, not as a replacement for classical approaches for diversity measurements from microbial communities where sequence variants (ASVs or OTUs) are used as observations. K-mer counting offers several advantages:

- k-mer counting allows rapid diversity comparison between samples, yielding ordinations that are well correlated with those derived from the phylogenetically aware beta diversity metric UniFrac (Fig. 1 and 2; Spearman *R* = 0.795, *P* < 0.001). Conventional non-phylogenetic beta diversity estimates are calculated based on species or sequence variant counts, which effectively treat all features as being fully independent and equidistant from one another. This has been an advantage of phylogenetically aware methods, which compare samples based on the amount of genetic diversity present in each sample, e.g., the fraction of branch length in a phylogeny that two samples have in common (3). k-mer counting offers this same advantage, comparing samples based on subsequence-level diversity (i.e., shared or dissimilar k-mers) while avoiding the high computational cost and challenges of accurate phylogeny estimation. This corresponds to the findings of Zhai and Fukuyama (20), who also found that Euclidean distances based on k-mer spectra of whole metagenome data correlated closely to the phylogenetic metric Rao’s quadratic entropy. Together, this suggests that k-mer-based distance metrics are a powerful tool for diversity measurements from both marker gene and metagenome data sets, which incorporate pseudo-phylogenetic information into diversity measurements as well as supervised learning analyses.
- Runtime is significantly less than phylogeny estimation techniques (Fig. 8). Nevertheless, k-mer counting is not intended as a phylogenetic estimate or replacement of phylogeny estimation wherever accurate phylogenies are required. However, in the context of microbial community beta diversity metrics, the goal is frequently to differentiate samples based on overall genetic diversity, not to estimate accurate phylogenies *per se*. Hence, k-mer counting is useful for distinguishing samples based on sequence topology.
- This approach allows sequence-based alpha and beta diversity comparison between non-coding regions with high mutation rates and poor phylogenetic signal, e.g., ITS sequences, which are otherwise challenging to use with phylogenetically aware beta diversity techniques.
- This approach can be used with any standard diversity metric, providing a high degree of flexibility and compatibility with different experimental hypotheses. Hence, k-mer frequencies can be compared with both qualitative (e.g., Bray Curtis dissimilarity) and quantitative metrics (e.g., Jaccard distance). Moreover, in contrast with existing phylogenetically aware methods, it is compatible with compositionally aware beta diversity metrics (e.g., Aitchison distance).
- The method is totally reference-free, enabling use in untargeted analysis across a range of ecosystems and target sequences. In other words, no reference data are used when counting k-mers, eliminating the need for curated reference sequences, alignments, or phylogenies in this type of analysis. This contrasts with some phylogeny estimation approaches, including insertion-based methods (5), which often rely on a reference alignment or phylogeny for reliable placement, limiting use for some sequence domains (e.g., ITS sequences and many non-ribosomal domains).

However, k-mer counting has several limitations that should be considered when applying this method:

- This approach should be used to compare sequences from the same target region. Comparing k-mer frequencies from different marker genes or domains may not yield meaningful results, as the k-mer frequency is expected to differ between different genes and domains. On the other hand, k-mer counting has been demonstrated as an effective technique for comparing whole (meta)genomes (17–20), so remains scalable as long as the comparable methodology is used, and potentially would provide a means of integrating data sets (an application out of scope in the current work and will be explored in the future).
- k-mer counting as implemented here is not a replacement for phylogenetic techniques, i.e., when an accurate estimate of evolutionary relationships is required, though k-mer counting has been demonstrated to accurately reflect evolutionary distances between genome sequences (13). Further work would be needed to demonstrate such an application with short DNA marker genes.
- k-mer counting can significantly expand the feature space, as each original sequence is broken up into many constituent k-mers. For this reason, various parameters are exposed to the user to constrain the feature space by filtering k-mers based on the minimum or maximum number of observations; specifying a maximum number of k-mers; and using TF-IDF to rank k-mers by importance. As many k-mers will also be highly redundant between different sequence variants (especially for relatively highly conserved sequence domains), these parameters are recommended to reduce redundancy as well as computational demands when appropriate. Some limited testing was performed here to demonstrate the influence of these parameters (supplemental results), but further testing is warranted; ultimately, k-mer frequency distributions and filtering settings will depend on study characteristics and experimental needs.

Using different feature types (e.g., k-mers vs sequence variants) as observations for diversity measurements requires some adjustments to interpretation, with complementary advantages when using these alongside classical approaches. For example, richness measurements based on ASV/OTU or species counts can be interpreted as the number of unique sequence variants or species observed in a sample; k-mer richness, on the other hand, relates to the amount of genetic diversity present. Thus, a sample with a low number of phylogenetically diverse species will yield higher k-mer richness and entropy than a sample with a large number of closely related species (Fig. S8), and measuring ASV/OTU/species diversity alongside k-mer diversity can yield a nuanced perspective on community composition.

Similarly, k-mer counting prior to beta diversity measurements allows distance/dissimilarity measurements to be adjusted by the amount of genetic relatedness (k-mer signatures) shared between those communities. Higher distances should then be interpreted as a higher degree of genetic diversity between samples; e.g., high Jaccard distance on k-mer counts would indicate that a low proportion of k-mers are shared between those samples. Classical beta diversity measurements based on ASV/OTU/species composition do not account for this, and hence distance/dissimilarity measurements can be exaggerated when unique variants (e.g., closely related strains that are differentiated by single or a few nucleotide polymorphisms) are present in one sample or the other (Fig. S10). This can be a strength when detection of subtle differences is needed, e.g., strain turnover events, but it can also skew interpretations when not considered alongside metrics that are informed by genetic diversity (e.g., UniFrac or k-mer-based metrics). The latter methods, on the other hand, have the advantage of moderating this effect, and instead prioritize unique genetic variants that drive differences between samples; this can lead to sensitive detection of genetically disparate variants that drive beta diversity differences, even when present in low frequency (Fig. S10). This interpretation is only possible when contrasting beta diversity results from multiple different metrics (Fig. S10). Hence, using classical approaches alongside k-mer-based (or phylogenetically aware) methods can lead to nuanced insight into the factors driving beta diversity observations, for inferring the degree to which genetically distinct clades explain variations in community composition.

Some additional caveats should be considered when using k-mer counting for diversity estimates. Regardless of whether ASVs/OTUs or k-mers are used as features, diversity metrics are highly sensitive to sampling effort (i.e., sequencing depth), and so rarefying or otherwise normalizing input data to control for differences in sequencing depth is needed to avoid bias (supplemental results, Fig. S2). This should be done at the level of sequence counts to control for different sequence depths before counting k-mers. As with conventional ASV/OTU-based diversity metrics, aggressive filtering will lead to a loss of sensitivity by removing rare variants (and by extension their k-mer signatures) from the data set. If desired, k-mer counts can also be controlled with the max_features parameter, though this is used rather for limiting the number of k-mers retrieved (e.g., to reduce computational costs associated with very large feature spaces) or for selection purposes (e.g., to retain only the most frequent). Filtering k-mers by prevalence and abundance (e.g., with the min_df and max_df parameters), as well as TF-IDF term weighting, offer different options for selecting k-mers, but these parameters should be used and interpreted with caution, especially when used prior to alpha diversity estimates, as estimates are then based off of a selection of features, not the full count. k-mer distribution and selection are likely to be impacted by study-specific characteristics and experimental needs, and investigators may need to adjust these parameters to optimize for a given experimental setting.

Another caveat is the selection of a suitable k-mer length, as this impacts methodological performance and interpretation to some extent. The default k-mer length selected here was *k* = 16, and it appears to work well across the large, diverse sample types included in these studies (the Earth Microbiome Project and Global Soil Mycobiome), so most likely generalizes to many study settings. Nevertheless, some researchers may wish to experiment with a few k-mer lengths to see how this impacts their results. In these cases, it is strongly recommended that they do so on a strictly qualitative basis, e.g., based on visual inspection of ordination plots, and to avoid statistical testing at this stage, as done in this study. This is recommended to avoid “*P*-hacking” to choose a solution that gives the best possible statistical result. Other strategies, e.g., testing on a subset of the data or an external data set from a similar population, may also be solutions to statistically optimize these settings prior to application in a full-sized study.

As k-mer counting yields potentially many unique k-mers from a given sequence, caution is needed during pre-processing and interpretation of results based on k-mer frequencies. This is because the k-mer frequencies generated from noisy or outlier sequences may be expected to amplify differences between samples. Naturally, such amplification is welcome and instructive if the k-mers derive from genuine sequence variants, but if these are introduced by, e.g., contaminants, erroneous, or off-target sequences they could yield spurious results, and careful pre-processing is required (as with any method). Alpha diversity and unweighted diversity metrics will be most sensitive to such errors, as will supervised learning techniques if these noisy k-mers correlate with a specific sample class. Comparing k-mer frequencies from different genetic targets (e.g., V4 vs V3-V4 domains of the 16S rRNA gene) could also yield such spurious results, as the total k-mer count and diversity will differ between targets. Hence, k-mer counting on its own is not a solution for comparing data from different genetic targets, e.g., in a multi-study comparison, unless it is paired with appropriate pre-processing steps to standardize inputs: e.g., by using closed-reference OTU clustering/read mapping to full-length reference genes (e.g., full-length 16S rRNA genes) so that the sequence templates are standardized to the same region prior to k-mer counting.

In conclusion, k-mer counting appears to be a suitable and efficient strategy for feature processing prior to diversity estimation as well as supervised learning in microbiome surveys. This allows the incorporation of subsequence-level information into diversity estimation without the computational cost of pairwise sequence alignment and is theoretically compatible with any classical (non-phylogenetic) diversity metric, provided that any method assumptions are met. k-mer-based diversity metrics demonstrated close correspondence with weighted and unweighted UniFrac metrics and Faith’s PD across sample types in the global-scale EMP data set, suggesting that k-mer frequencies yield approximately similar information to these phylogenetic metrics. K-mer counting is proposed as a complementary approach to classical diversity estimates for feature processing prior to diversity estimation and supervised learning analyses, enabling large-scale reference-free profiling of microbiomes in biogeography, ecology, and biomedical data.

## Data Availability

The EMP and GSM data sets are publicly available as described in the text. The EMP files used in this study, as well as a subset of the GSM data set used here (selected as described in the text), are available on Zenodo (https://zenodo.org/records/13304967). A method for k-mer counting is implemented in the QIIME 2 plugin q2-kmerizer (https://github.com/bokulichlab/q2-kmerizer).

## SUPPLEMENTAL MATERIAL

Supplemental Material msystems.01550-24-s0001.pdf Supplemental figures, tables, results, and methodology.

ASM does not own the copyrights to Supplemental Material that may be linked to, or accessed through, an article. The authors have granted ASM a non-exclusive, world-wide license to publish the Supplemental Material files. Please contact the corresponding author directly for reuse.

## References

[1] Thompson LR, Sanders JG, McDonald D, Amir A, Ladau J, Locey KJ, Prill RJ, Tripathi A, Gibbons SM, Ackermann G, et al.. 2017. A communal catalogue reveals Earth’s multiscale microbial diversity. Nature New Biol 551:457–463. doi:10.1038/nature24621 PMC6192678 29088705

[2] Tedersoo L, Mikryukov V, Anslan S, Bahram M, Khalid AN, Corrales A, Agan A, Vasco-Palacios A-M, Saitta A, Antonelli A, et al.. 2021. The global soil mycobiome consortium dataset for boosting fungal diversity research. Fungal Divers 111:573–588. doi:10.1007/s13225-021-00493-7

[3] Lozupone C, Knight R. 2005. UniFrac: a new phylogenetic method for comparing microbial communities. Appl Environ Microbiol 71:8228–8235. doi:10.1128/AEM.71.12.8228-8235.2005 16332807 PMC1317376

[4] Schloss PD. 2008. Evaluating different approaches that test whether microbial communities have the same structure. ISME J 2:265–275. doi:10.1038/ismej.2008.5 18239608

[5] Janssen S, McDonald D, Gonzalez A, Navas-Molina JA, Jiang L, Xu ZZ, Winker K, Kado DM, Orwoll E, Manary M, Mirarab S, Knight R. 2018. Phylogenetic placement of exact amplicon sequences improves associations with clinical information. mSystems 3:e00021-18. doi:10.1128/mSystems.00021-18 29719869 PMC5904434

[6] Schoch CL, Seifert KA, Huhndorf S, Robert V, Spouge JL, Levesque CA, Chen W, Null N, Bolchacova E, Voigt K. 2012. Nuclear ribosomal internal transcribed spacer (ITS) region as a universal DNA barcode marker for Fungi. Proc Natl Acad Sci U S A 109:6241–6246. doi:10.1073/pnas.1117018109 22454494 PMC3341068

[7] Fouquier J, Rideout JR, Bolyen E, Chase J, Shiffer A, McDonald D, Knight R, Caporaso JG, Kelley ST. 2016. Ghost-tree: creating hybrid-gene phylogenetic trees for diversity analyses. Microbiome 4:1–10. doi:10.1186/s40168-016-0153-6 26905735 PMC4765138

[8] Simmons MP, Freudenstein JV. 2003. The effects of increasing genetic distance on alignment of, and tree construction from, rDNA internal transcribed spacer sequences. Mol Phylogenet Evol 26:444–451. doi:10.1016/s1055-7903(02)00366-4 12644403

[9] Wang Q, Garrity GM, Tiedje JM, Cole JR. 2007. Naive Bayesian classifier for rapid assignment of rRNA sequences into the new bacterial taxonomy. Appl Environ Microbiol 73:5261–5267. doi:10.1128/AEM.00062-07 17586664 PMC1950982

[10] Bokulich NA, Kaehler BD, Rideout JR, Dillon M, Bolyen E, Knight R, Huttley GA, Gregory Caporaso J. 2018. Optimizing taxonomic classification of marker-gene amplicon sequences with QIIME 2’s q2-feature-classifier plugin. Microbiome 6:90. doi:10.1186/s40168-018-0470-z 29773078 PMC5956843

[11] Ziemski M, Wisanwanichthan T, Bokulich NA, Kaehler BD. 2021. Beating naive bayes at taxonomic classification of 16S rRNA gene sequences. Front Microbiol 12:644487. doi:10.3389/fmicb.2021.644487 34220738 PMC8249850

[12] Wood DE, Lu J, Langmead B. 2019. Improved metagenomic analysis with Kraken 2. Genome Biol 20:257. doi:10.1186/s13059-019-1891-0 31779668 PMC6883579

[13] Morgenstern B, Zhu B, Horwege S, Leimeister CA. 2015. Estimating evolutionary distances between genomic sequences from spaced-word matches. Algorithms Mol Biol 10:5. doi:10.1186/s13015-015-0032-x 25685176 PMC4327811

[14] Compeau PEC, Pevzner PA, Tesler G. 2011. How to apply de Bruijn graphs to genome assembly. Nat Biotechnol 29:987–991. doi:10.1038/nbt.2023 22068540 PMC5531759

[15] Woloszynek S, Zhao Z, Chen J, Rosen GL. 2019. 16S rRNA sequence embeddings: meaningful numeric feature representations of nucleotide sequences that are convenient for downstream analyses. PLoS Comput Biol 15:e1006721. doi:10.1371/journal.pcbi.1006721 30807567 PMC6407789

[16] Asgari E, Garakani K, McHardy AC, Mofrad MRK. 2018. MicroPheno: predicting environments and host phenotypes from 16S rRNA gene sequencing using a k-mer based representation of shallow sub-samples. Bioinformatics 34:i32–i42. doi:10.1093/bioinformatics/bty296 29950008 PMC6022683

[17] Dubinkina VB, Ischenko DS, Ulyantsev VI, Tyakht AV, Alexeev DG. 2016. Assessment of k-mer spectrum applicability for metagenomic dissimilarity analysis. BMC Bioinformatics 17:1–11. doi:10.1186/s12859-015-0875-7 26774270 PMC4715287

[18] Choi I, Ponsero AJ, Bomhoff M, Youens-Clark K, Hartman JH, Hurwitz BL. 2019. Libra: scalable k-mer-based tool for massive all-vs-all metagenome comparisons. Gigascience 8:giy165. doi:10.1093/gigascience/giy165 30597002 PMC6354030

[19] Pierce NT, Irber L, Reiter T, Brooks P, Brown CT. 2019. Large-scale sequence comparisons with sourmash. F1000Res 8:1006. doi:10.12688/f1000research.19675.1 31508216 PMC6720031

[20] Zhai H, Fukuyama J. 2023. A convenient correspondence between k-mer-based metagenomic distances and phylogenetically-informed β-diversity measures. PLoS Comput Biol 19:e1010821. doi:10.1371/journal.pcbi.1010821 36608056 PMC9879504

[21] Buitinck L, Louppe G, Blondel M, Pedregosa F, Mueller A, Grisel O, Niculae V, Prettenhofer P, Gramfort A, Grobler J, Layton R, Vanderplas J, Joly A, Holt B, Varoquaux G. 2013. API design for machine learning software: experiences from the scikit-learn project.

[22] Harris CR, Millman KJ, van der Walt SJ, Gommers R, Virtanen P, Cournapeau D, Wieser E, Taylor J, Berg S, Smith NJ, et al.. 2020. Array programming with NumPy. Nature New Biol 585:357–362. doi:10.1038/s41586-020-2649-2 PMC7759461 32939066

[23] Sparck Jones K. 1972. A statistical interpretation of term specificity and its application in retrieval. J Doc 28:11–21. doi:10.1108/eb026526

[24] Bolyen E, Rideout JR, Dillon MR, Bokulich NA, Abnet CC, Al-Ghalith GA, Alexander H, Alm EJ, Arumugam M, Asnicar F, et al.. 2019. Reproducible, interactive, scalable and extensible microbiome data science using QIIME 2. Nat Biotechnol 37:852–857. doi:10.1038/s41587-019-0209-9 31341288 PMC7015180

[25] Robeson MS, O’Rourke DR, Kaehler BD, Ziemski M, Dillon MR, Foster JT, Bokulich NA. 2021. RESCRIPt: reproducible sequence taxonomy reference database management. PLoS Comput Biol 17:e1009581. doi:10.1371/journal.pcbi.1009581 34748542 PMC8601625

[26] Anderson MJ. 2017. Permutational multivariate analysis of variance (PERMANOVA), p 1–15. In Wiley StatsRef: statistics reference online. John Wiley & Sons, Ltd.

[27] Bokulich NA, Dillon MR, Zhang Y, Rideout JR, Bolyen E, Li H, Albert PS, Caporaso JG. 2018. Q2-longitudinal: longitudinal and paired-sample analyses of microbiome data. mSystems 3:e00219-18. doi:10.1128/msystems.00219-18 PMC6247016 30505944

[28] Virtanen P, Gommers R, Oliphant TE, Haberland M, Reddy T, Cournapeau D, Burovski E, Peterson P, Weckesser W, Bright J, et al.. 2020. SciPy 1.0 contributors. Nat Methods 17:352. doi:10.1038/s41592-020-0772-5 32094914 PMC7056641

[29] Waskom ML. 2021. Seaborn: statistical data visualization. JOSS 6:3021. doi:10.21105/joss.03021

[30] Gower JC. 1975. Generalized procrustes analysis. Psychometrika 40:33–51. doi:10.1007/BF02291478

[31] Rousseeuw PJ. 1987. Silhouettes: a graphical aid to the interpretation and validation of cluster analysis. J Comput Appl Math 20:53–65. doi:10.1016/0377-0427(87)90125-7

[32] Breiman L. 2001. Random forests. Mach Learn 45:5–32. doi:10.1023/A:1010933404324

[33] Bokulich N, Dillon M, Bolyen E, Kaehler B, Huttley G, Caporaso J. 2018. Q2-sample-classifier: machine-learning tools for microbiome classification and regression. JOSS 3:934. doi:10.21105/joss.00934 PMC6759219 31552137

[34] Katoh K, Misawa K, Kuma K, Miyata T. 2002. MAFFT: a novel method for rapid multiple sequence alignment based on fast Fourier transform. Nucleic Acids Res 30:3059–3066. doi:10.1093/nar/gkf436 12136088 PMC135756

[35] Price MN, Dehal PS, Arkin AP. 2010. FastTree 2--approximately maximum-likelihood trees for large alignments. PLoS One 5:e9490. doi:10.1371/journal.pone.0009490 . 20224823 PMC2835736

