# Supplementary material for: Integrating sequence composition information into microbial diversity analyses with k-mer frequency counting
Source: mSystems. 2025 Feb 20;10(3):e01550-24. doi: 10.1128/msystems.01550-24 (PMC11915819; doi:10.1128/msystems.01550-24)
Supplement: Supplemental Material — Supplemental figures, tables, results, and methodology. [file msystems.01550-24-s0001.pdf]

Supplemental Material for: Integrating sequence composition information into microbial diversity analyses with k-mer frequency counting

Supplementary Results

Parameter testing

Several small benchmarks were conducted to examine the influence of various sampling constraints on k-mer-based beta diversity estimates: sampling depth (i.e., rarefying prior to k-mer counting), max features (i.e., the feature count kept after k-mer counting), TF-IDF normalization, and abundance-based filtering after k-mer counting. This is not intended as an all-comprehensive benchmark, as several results will depend on study-specific characteristics, e.g., the genetic target and the composition of the samples in a given experiment.

As can be expected, sampling depth (i.e., single rarefaction to normalize samples by sequencing depth) impacts k-mer-based beta diversity estimates, though the effect is relatively small. PCoA plots (Figure S2) and distance-distance plots and Mantel tests (Figure S3) demonstrate similar distances and clustering patterns from depths of 500 to 8000. A very low sampling depth (d=500) led to a distorted ordination and lower Mantel correlation to the highest depth (Bray-Curtis PERMANOVA R = 0.924), but still retained the overall clustering pattern and similar % variance explained by PERMANOVA ($P < 0.001$, R2 = 0.418) compared to d=8000 ($P < 0.001$, R2 = 0.408). Nevertheless, as for ASVs/OTUs, higher sampling depths are clearly beneficial and necessary for fully characterizing the diversity present in a given sample. This mini-benchmark was performed simply for instructive purposes to test how robust k-mer-based beta diversity estimates are to sampling depth; the important outcome is the observation that ordination results and distance-distance comparisons are not wildly different even across a 16-fold difference in sampling depth variations.

Similarly, abundance-based k-mer filtering impacts beta diversity results (Figures S4-S5). Scikit-learn's CountVectorizer method exposes a max_features parameter, which constrains the feature space to keep the *m* most abundant k-mers after k-mer counting. Intuitively, the max_features parameter should be set to "None" in cases where a moderate k-mer count is expected, or when computational resources are not limiting, so that maximal information is kept. However, it may be beneficial to set this parameter in some cases, e.g., to automatically filter out low-abundant, noisy k-mers, and to constrain the k-mer space to a tractable limit (as very large k-mer counts, e.g., in highly diversity datasets, could lead to issues with computational performance). This parameter deserves further examination (outside of the scope of the present study), but a quick benchmark was done here to examine the basic relationships between this parameter and beta diversity estimates. At high max feature counts (e.g., 10000 vs. 20000), beta diversity estimates were closely correlated, but lower max feature counts led to progressively degraded performance (Figures S4-S5). Further testing is warranted to optimize this parameter in study-specific settings.

K-mer-based diversity measurements on synthetic data

Alpha diversity simulation. In addition to testing k-mer counting on the EMP and GSM datasets, synthetic data were used to test the performance of k-mer counting on datasets with defined characteristics. Alpha and beta diversity were calculated using synthetic datasets to evaluate the impact of simulated data characteristics (feature type, distribution, genetic diversity) on diversity estimates. For the alpha diversity evaluation, numpy (1) was used to simulate feature frequencies of defined feature richnesses (*r*) between 1 and 1000, by drawing feature frequencies from an exponential distribution: $f(n) = 10^{nx}$, Where *n* is a factor modifying the exponential slope of the distribution. Frequency distributions were generated for values of *x* between 0 (i.e., even distribution) and 10 (i.e., high uneven) at each richness level. Each frequency distribution was then annotated with taxonomies randomly drawn from the SILVA small subunit ribosomal rRNA gene database (2) within different taxonomic clades to simulate collections of sequences with low heterogeneity (i.e., all sequences are drawn from within the same genus, here using *Lactobacillus*), moderate heterogeneity (random sequences drawn from the phylum Bacillota), or high heterogeneity (totally random sequences from within the collection). K-mer frequencies were calculated from the corresponding sequences as described above and entropy (i.e., Shannon's *H* index) of each simulated community was calculated using scipy (3).

As expected, Shannon entropy (based on feature counts) is a function of both richness and evenness (Figure S8B). Observed k-mer counts are slightly non-linearly associated with observed ASVs (Figure S8C,D,E; note that observed kmers are shown on a logarithmic scale in Figure S8), corresponding to the observations made with the EMP and GSM datasets. As expected, kmer entropy (Figure S8D,F,H) is considerably higher than ASV entropy (Figure S8B), and both observed k-mers and k-mer entropy are higher in simulated communities with increasing levels of taxonomic representation (Figure S8C-H). This demonstrates the utility of k-mer-based alpha diversity metrics as complementary to conventional alpha diversity measurements, as the k-mer-based metrics are a measure of genetic heterogeneity, and are responsive to increasing genetic diversity within the community.

Beta diversity simulation. For the beta diversity evaluation, frequency distributions of 100 "samples" each consisting of 70-100 unique ASVs were simulated using numpy.random.randit and annotated with different taxonomic labels to represent five distinct groups of samples with varying degrees of heterogeneity (compositions shown in Figure S9):

A. Beta diversity of "typical" communities of moderate diversity, consisting primarily of the genus *Lactobacillus*, other *Lactobacillales*, and genus *Acetobacter*, lower abundances of the genera *Bacillus, Staphylococcus*, and family *Enterobacteriaceae*, and random other bacterial sequences drawn at very low frequencies (Supplementary Figure S9)
B. Identical ASVs as in A, but with slightly adjusted distributions to simulate communities with small distances/dissimilarities to A.
C. "Alternative" ASVs: The same feature distributions as in A, but with different ASVs drawn from the same clades (such that the ASVs are genetically very similar but are distinct from those in A). This is done to demonstrate the advantages of **conventional metrics** (which are phylogenetically unaware and treat all features as equally unrelated regardless of their genetic similarity) to sensitively detect differences between samples based on presence of unique features, e.g., of sub-species variants.
D. "Rare species": Same as A, but with many additional ASVs (from random bacterial lineages) added at a very low frequency. This is done to demonstrate the ability of unweighted metrics to detect differences between samples based on the presence/absence of rare features.
E. "Rare distant" ASVs: Same as A, but with the addition of two Archaea species (one randomly selected *Nanoarchaeum* sp. and one randomly selected *Halobacterium* sp.) at low frequencies. This

is done to simulate the ability of metrics that take genetic information into account (k-mer-based and UniFrac metrics) to weight their distances/dissimilarities by genetic relatedness, detecting differences between samples based on the presence of distinct clades.

These samples are not necessarily intended to represent natural communities, but to simulate different community characteristics that demonstrate the strengths of the conventional, phylogeny-aware, and kmer-based beta diversity metrics. These collections were chosen to represent (a-b) “typical” community compositions with low-to-moderate diversity; (c) to highlight the advantages of conventional diversity metrics (i.e., non-phylogeny-aware and based on ASV frequencies) to identify differences driven by distinct features that have low genetic heterogeneity (e.g., the scenario when beta diversity differences are driven by strain-level variation); (d) to demonstrate the advantages of unweighted metrics (regardless of feature type) for detecting differences driven by the presence of rare features; and (e) to demonstrate the advantages of kmer-based and phylogeny-aware metrics to identify differences that are driven by the presence of genetically distant clades in a community. After simulation, the simulated observation matrix was subsampled at 9000 observations per sample and beta diversity was measured and statistically tested as described above. For UniFrac metrics, sequences were aligned with mafft (4) followed by phylogeny estimation with raxml (5).

All metrics could detect significant differences in the simulated communities (Figure S10), including differences between communities A and B (PERMANOVA P-values range from 0.001 to 0.022; Tables S8-9). However, PCoA ordinations reveal specific characteristics and strengths of each metric (Figure S10). As expected, the conventional metrics (ASV-based Jaccard and Bray-Curtis) can sensitively discriminate between communities that contain ASVs that are genetically similar but belong to distinct accessions (i.e., strains). This is useful in scenarios where subtle differences in communities are expected, e.g., due to turnover of individual strains within a species; but it could be disadvantageous in other scenarios when such subtle differences are not functionally relevant, as this can lead to exaggerated differences between these communities. Also as expected, unweighted metrics (ASV Jaccard, k-mer Jaccard, and unweighted UniFrac) were most sensitive at differentiating the “rare species” communities, highlighting a classical use case for testing unweighted metrics alongside weighted metrics. Finally, k-mer-based and UniFrac metrics were most able to differentiate the “rare distant” communities (containing two genetically dissimilar Archaea ASVs added at low frequencies), and to varying degrees. UniFrac shows the highest % variation explained in the first two principal coordinates (Figure S10), and higher PERMANOVA effect sizes when comparing this group to the other groups (Table S8), followed by k-mer-based metrics. Thus, UniFrac may have a possible advantage from utilizing actual phylogenetic information, though this is a double-edged sword as it is also sensitive to tree topology, including in cases where poor phylogenies are created, e.g., from short sequences, problematic alignments, or outlier clades (6). K-mer diversity, on the other hand, shows slightly more sensitivity to the presence of genetically similar variants (Figure S10 and Table S8; compare groups A and C). Hence, there may also be complementarity in using these methods alongside each other.

Supplementary References

1. Harris CR, Millman KJ, van der Walt SJ, Gommers R, Virtanen P, Cournapeau D, Wieser E, Taylor J, Berg S, Smith NJ, Kern R, Picus M, Hoyer S, van Kerkwijk MH, Brett M, Haldane A, Del Río JF, Wiebe M, Peterson P, Gérard-Marchant P, Sheppard K, Reddy T, Weckesser W, Abbasi H, Gohlke C, Oliphant TE. 2020. Array programming with NumPy. Nature 585:357–362.

2. Quast C, Pruesse E, Yilmaz P, Gerken J, Schweer T, Yarza P, Peplies J, Glöckner FO. 2013. The SILVA

ribosomal RNA gene database project: improved data processing and web-based tools. Nucleic Acids Res 41:D590–6.

3. Virtanen P, Gommers R, Oliphant TE, Haberland M, Reddy T, Cournapeau D, Burovski E, Peterson P, Weckesser W, Bright J, van der Walt SJ, Brett M, Wilson J, Millman KJ, Mayorov N, Nelson ARJ, Jones E, Kern R, Larson E, Carey CJ, Polat İ, Feng Y, Moore EW, VanderPlas J, Laxalde D, Perktold J, Cimrman R, Henriksen I, Quintero EA, Harris CR, Archibald AM, Ribeiro AH, Pedregosa F, van Mulbregt P, SciPy 1.0 Contributors. 2020. Author Correction: SciPy 1.0: fundamental algorithms for scientific computing in Python. Nat Methods 17:352.

4. Katoh K, Misawa K, Kuma K-I, Miyata T. 2002. MAFFT: a novel method for rapid multiple sequence alignment based on fast Fourier transform. Nucleic Acids Res 30:3059–3066.

5. Stamatakis A. 2014. RAxML version 8: a tool for phylogenetic analysis and post-analysis of large phylogenies. Bioinformatics 30:1312–1313.

6. Janssen S, McDonald D, Gonzalez A, Navas-Molina JA, Jiang L, Xu ZZ, Winker K, Kado DM, Orwoll E, Manary M, Mirarab S, Knight R. 2018. Phylogenetic Placement of Exact Amplicon Sequences Improves Associations with Clinical Information. mSystems https://doi.org/10.1128/msystems.00021-18.

Tables

Table S1. Mantel test correlations and Procrustes goodness-of-fit (M^2) between different distance metrics and feature processing methods on the EMP dataset. K-mers size = 16.

		**Mantel Spearman *R***	**p-value**	**Procrustes M^2**	**p-value**
ASV Jaccard	Unweighted UniFrac	0.551	0.001	0.354	0.001
ASV Jaccard	K-mer Jaccard	0.656	0.001	0.193	0.001
Kmer Jaccard	Unweighted UniFrac	0.719	0.001	0.096	0.001
ASV Bray Curtis	K-mer Bray Curtis	0.542	0.001	0.228	0.001
ASV Bray Curtis	Weighted UniFrac	0.405	0.001	0.475	0.001
Kmer Bray Curtis	Weighted UniFrac	0.795	0.001	0.203	0.001

Table S2. PERMANOVA tests for differentiation by EMPO 3 class (EMP dataset). K-mers size = 16.

**Metric**	**Method**	**R^2**	**P-value**
Aitchison	ASV	0.18	0.001
	K-mer	0.35	0.001
	K-mer TF-IDF	0.34	0.001
Bray Curtis	ASV	0.22	0.001
	K-mer	0.34	0.001
	K-mer TF-IDF	0.33	0.001
Jaccard	ASV	0.15	0.001
	K-mer	0.31	0.001
	K-mer TF-IDF	0.31	0.001
Unweighted UniFrac	ASV	0.29	0.001
Weighted UniFrac	ASV	0.38	0.001

Table S3. Summary of pairwise PERMANOVA tests between each EMPO 3 class (EMP dataset) and comparison between each distance metric and type of feature processing method. K-mers size = 16.

Metric 1	Type 1	Metric 2	Type 2	Diff(r2)*	Statistic	P-value
**Aitchison**	**ASV**	**Aitchison**	Kmer	0.110	1.0	< 0.001
			Kmer TF-IDF	0.098	1.0	< 0.001
	**K-mer**	**Aitchison**	Kmer TF-IDF	-0.012	290.0	< 0.001
**Bray-Curtis**	**ASV**	**Bray-Curtis**	Kmer TF-IDF	0.080	4.0	< 0.001
			Kmer TF-IDF	0.073	1.0	< 0.001
		**Weighted UniFrac**	ASV	0.109	22.0	< 0.001
	**K-mer**	**Bray-Curtis**	Kmer TF-IDF	-0.007	419.0	< 0.001
		**Weighted UniFrac**	ASV	0.029	824.0	< 0.001
	**K-mer**	**Weighted UniFrac**	ASV	0.036	642.0	< 0.001
**Jaccard**	**ASV**	**Jaccard**	Kmer	0.105	0.0	< 0.001
			Kmer TF-IDF	0.105	0.0	< 0.001
		**Unweighted UniFrac**	ASV	0.079	0.0	< 0.001
	**K-mer**	**Jaccard**	Kmer TF-IDF	0.000	2730.0	0.847
		**Unweighted UniFrac**	ASV	-0.025	1000.0	< 0.001
	**K-mer TF-IDF**	**Unweighted UniFrac**	ASV	-0.025	1000.0	< 0.001

*Diff(r2): mean difference in PERMANOVA R2 value. Mean R2 for metric/type 2 - R2 for metric/type 1. A positive difference indicates that R2 values for metric/type 2 are consistently higher than those for metric/type 1; a negative value indicates the opposite relationship.

Table S4. Procrustes goodness-of-fit (M2) scores by k-mer length (*k*) and metric between k-mer-based distances and UniFrac distances.

	Method*	
k	**Unweighted****	**Weighted*****
**3**	0.987	0.439
**5**	0.587	0.239
**7**	0.205	0.186
**9**	0.211	0.174
**12**	0.209	0.180
**16**	0.211	0.187
**24**	0.245	0.228
**32**	0.217	0.321

*For all tests P values = 0.001
**Unweighted = Jaccard distance on k-mers vs. unweighted UniFrac distance on ASVs.
***Weighted = Bray Curtis distance on k-mers vs. weighted UniFrac distance on ASVs.

Table S5. Correlations between alpha diversity estimates in the Earth Microbiome Project dataset. K-mer size = 16.

		**Pearson *R***	**P-value**	**Spearman *R***	**P-value**
Observed ASVs	Observed k-mers	0.981	< 0.001	0.991	< 0.001
Shannon ASVs	Shannon k-mers	0.988	< 0.001	0.986	< 0.001
Observed ASVs	Faith PD (ASVs)	0.980	< 0.001	0.990	< 0.001
Faith PD (ASVs)	Observed k-mers	0.977	< 0.001	0.986	< 0.001
Shannon ASVs	Faith PD (ASVs)	0.859	< 0.001	0.946	< 0.001
Faith PD (ASVs)	Shannon k-mers	0.844	< 0.001	0.944	< 0.001

Table S6. PERMANOVA tests for differentiation by biome type (GSM dataset). K-mer size = 16.

**Metric**	**Method**	**R^2**	**P-value**
Aitchison	ASV	0.06	0.001
	K-mer	0.26	0.001
	K-mer TF-IDF	0.26	0.001
Bray Curtis	ASV	0.06	0.001
	K-mer	0.18	0.001
	K-mer TF-IDF	0.19	0.001
Jaccard	ASV	0.04	0.001
	K-mer	0.27	0.001
	K-mer TF-IDF	0.27	0.001

Table S7. Summary of pairwise PERMANOVA tests between each biome type (GSM dataset) and comparison between each distance metric and type of feature processing method. K-mer size = 16.

**Metrics**	**Type 1**	**Type 2**	**Diff(r2)***	**Statistic**	**P-value**
**Aitchison**	**ASV**	**Kmer**	0.109	0.0	< 0.001
		**Kmer TF-IDF**	0.111	0.0	< 0.001
	**Kmer**	**Kmer TF-IDF**	0.002	74.0	0.157
**Bray-Curtis**	**ASV**	**Kmer**	0.067	0.0	< 0.001
		**Kmer TF-IDF**	0.070	0.0	< 0.001
	**Kmer**	**Kmer TF-IDF**	0.003	65.0	0.082
**Jaccard**	**ASV**	**Kmer**	0.121	0.0	< 0.001
		**Kmer TF-IDF**	0.121	0.0	< 0.001
	**Kmer**	**Kmer TF-IDF**	0.000	115.5	1.000

Table S8. PERMANOVA test results (R2 values) of pairwise comparisons between simulated communities. K-mer size = 16.

	Metric	Bray-Curtis	Jaccard	Weighted Unifrac	Unweighted Unifrac	Bray-Curtis	Jaccard
	**Type**	**ASV**	**ASV**	**ASV**	**ASV**	**K-mer**	**K-mer**
**Group A**	**Group B**						
A	B	0.051	0.037	0.152	0.053	0.089	0.040
A	C	0.197	0.130	0.072	0.097	0.119	0.102
A	D	0.037	0.256	0.082	0.446	0.057	0.403
A	E	0.053	0.073	0.240	0.309	0.069	0.131
B	C	0.169	0.112	0.177	0.063	0.142	0.082
B	D	0.055	0.233	0.112	0.426	0.090	0.369
B	E	0.053	0.062	0.218	0.290	0.088	0.117
C	D	0.192	0.260	0.118	0.437	0.128	0.381
C	E	0.193	0.152	0.281	0.335	0.158	0.177
D	E	0.068	0.287	0.229	0.585	0.087	0.453

Table S9. PERMANOVA test results (P values) of pairwise comparisons between simulated communities. K-mer size = 16.

	Metric	Bray-Curtis	Jaccard	Weighted Unifrac	Unweighted Unifrac	Bray-Curtis	Jaccard
	**Type**	**ASV**	**ASV**	**ASV**	**ASV**	**K-mer**	**K-mer**
**Group A**	**Group B**						
A	B	0.002	0.022	0.001	0.012	0.001	0.022
A	C	0.001	0.001	0.004	0.001	0.001	0.001
A	D	0.098	0.001	0.007	0.001	0.006	0.001
A	E	0.002	0.001	0.001	0.001	0.001	0.001
B	C	0.001	0.001	0.001	0.003	0.001	0.001
B	D	0.001	0.001	0.002	0.001	0.001	0.001
B	E	0.001	0.001	0.001	0.001	0.001	0.001
C	D	0.001	0.001	0.001	0.001	0.001	0.001
C	E	0.001	0.001	0.001	0.001	0.001	0.001
D	E	0.001	0.001	0.001	0.001	0.001	0.001

Figures

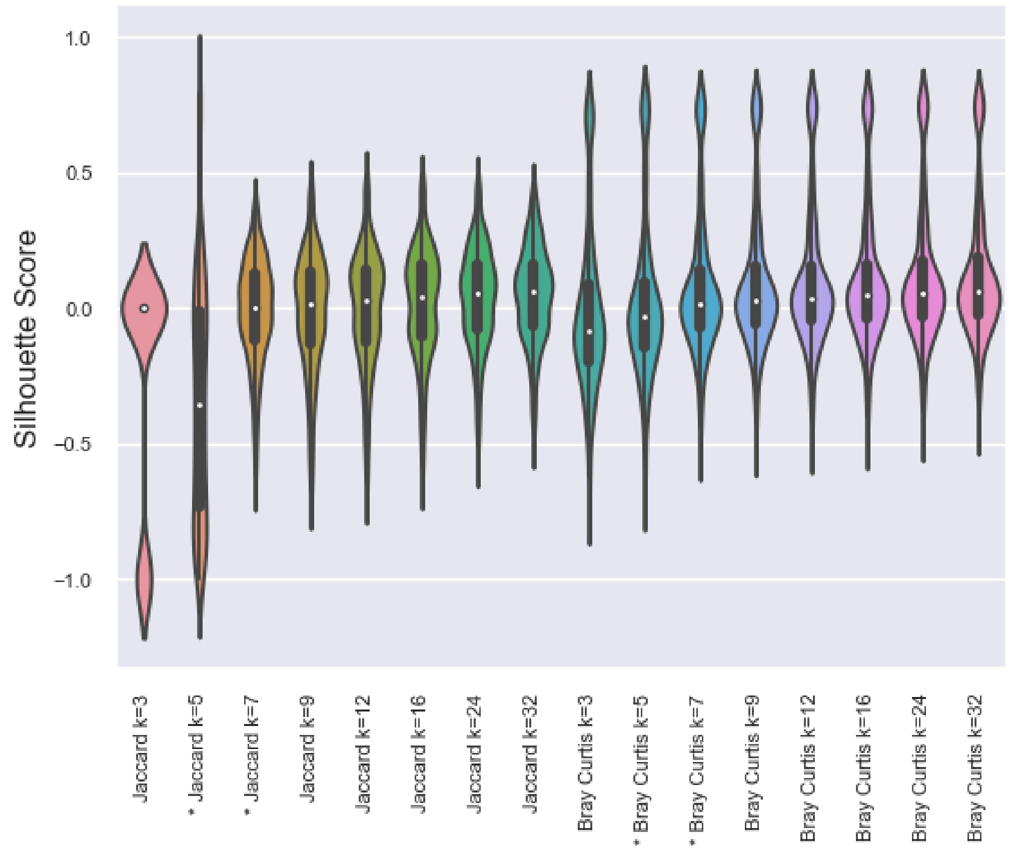

Figure S1. K-mer size influences sample clustering in the EMP dataset. Violin plots illustrate the silhouette coefficient distributions across all EMP samples, based on Jaccard distances (left) and Bray-Curtis dissimilarities (right) calculated from k-mer frequencies of different lengths of *k*. The Silhouette coefficient indicates the clustering quality; scores near 0 indicate overlapping clusters, +1 indicates perfect clustering by sample type, and -1 indicates that a sample is clustered incorrectly (i.e., it is clustered together with samples from a different group). Asterisks indicate that the distances/dissimilarities for a given value of *k* are significantly different from the preceding value of *k*.

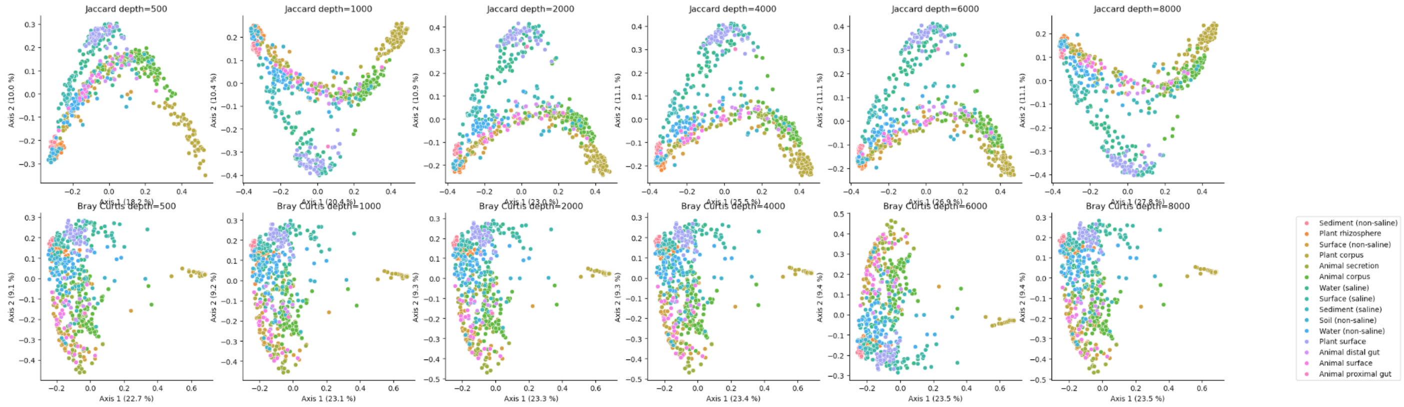

Figure S2. Sampling depth for rarefying impacts k-mer-based beta diversity estimates (k=16). Principal coordinate plots illustrate sample ordinations with different sampling depths.

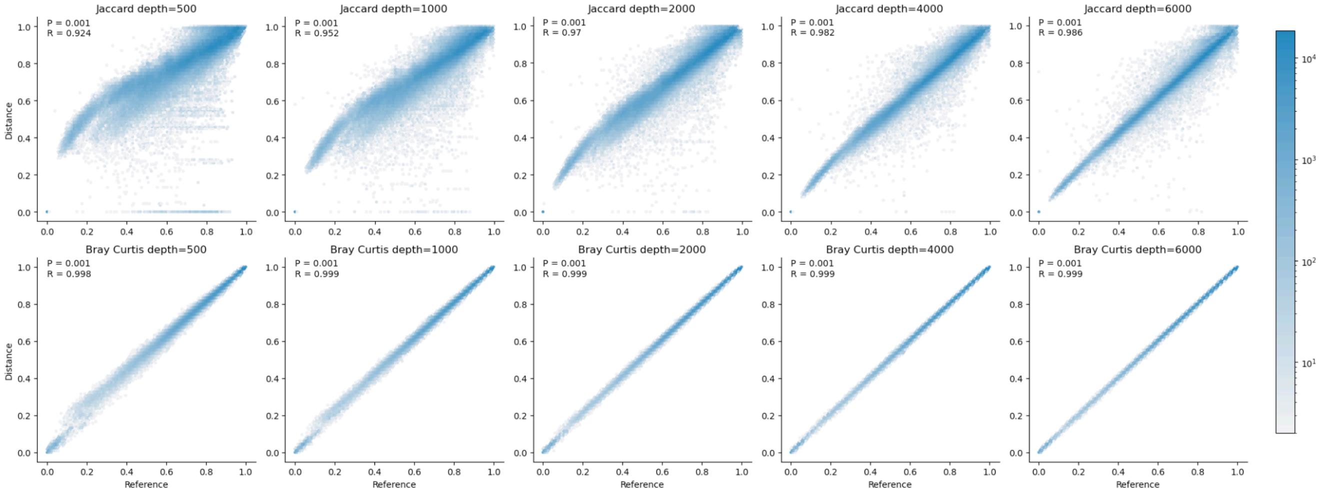

Figure S3. K-mer-based diversity metrics are closely correlated in the EMP dataset between sampling depths of d=500 and d=8000. Distance-distance correlation plots show the correlation between distances observed at d=8000 (the maximum depth tested) with depths from d=500 to d=6000. These results demonstrate that Jaccard distance and Bray-Curtis dissimilarity results are fairly robust to different sampling depths. K-mer size = 16.

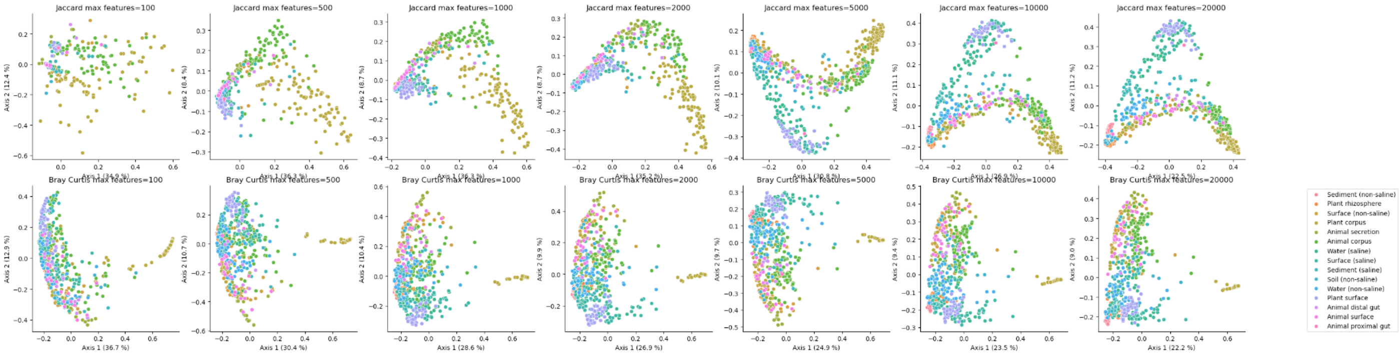

Figure S4. Abundance-based k-mer filtering impacts beta diversity ordination results. Principal coordinate plots illustrate sample ordinations with different max_feature counts (i.e., the number of k-mers kept by CountVectorizer, keeping the *m* most abundant k-mers). K-mer size = 16.

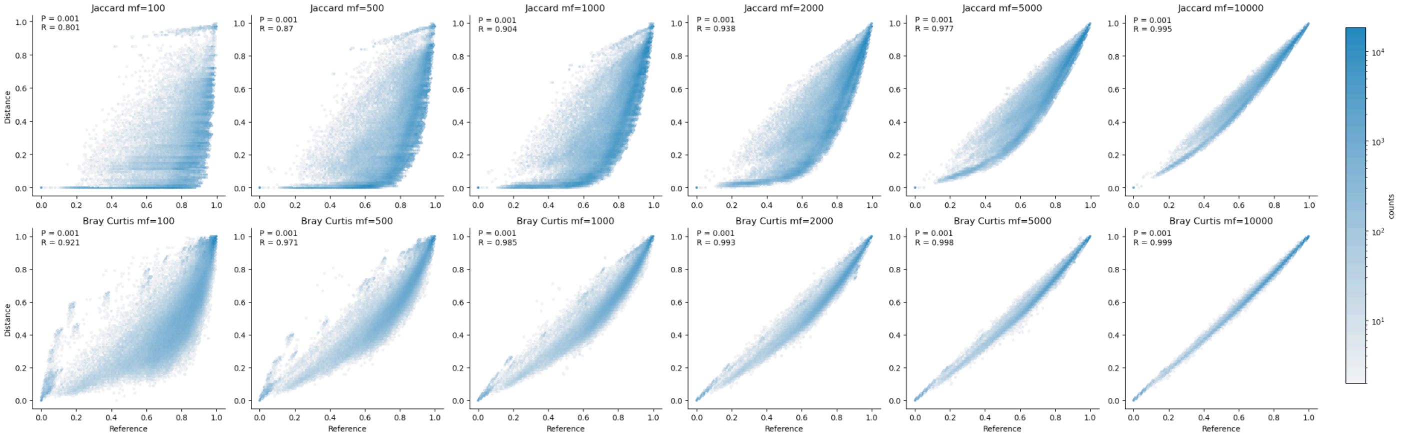

Figure S5. Abundance-based k-mer filtering impacts beta diversity distance/dissimilarity results. Distance-distance correlation plots show the correlation between distances observed at 20000 max features vs. between 100-10000 max features. These results demonstrate that Jaccard distance and Bray-Curtis dissimilarity results are impacted by stringent k-mer abundance filtering. K-mer size = 16.

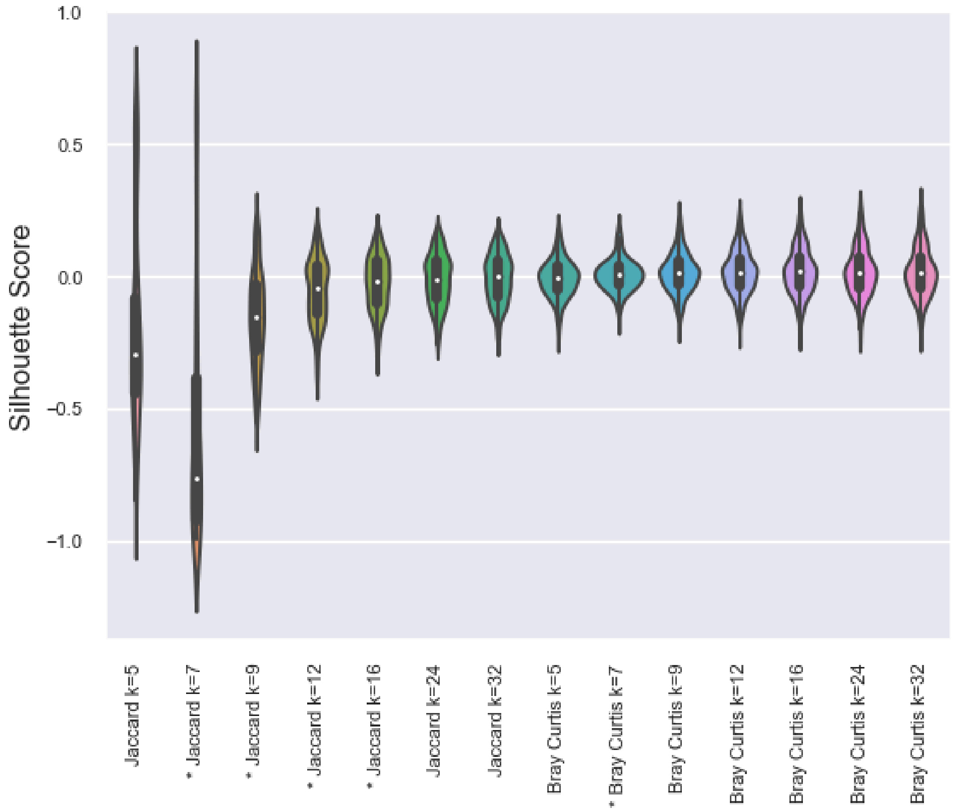

Figure S6. K-mer size influences sample clustering in the GSM dataset. Violin plots illustrate the silhouette coefficient distributions across all GSM samples, based on Jaccard distances (left) and Bray-Curtis dissimilarities (right) calculated from k-mer frequencies of different lengths of *k*. The Silhouette coefficient indicates the clustering quality; scores near 0 indicate overlapping clusters, +1 indicates perfect clustering by sample type, and -1 indicates that a sample is clustered incorrectly (i.e., it is clustered together with samples from a different group). Asterisks indicate that the distances/dissimilarities for a given value of *k* are significantly different (Wilcoxon rank-sum $P < 0.05$) from the preceding value of *k*.

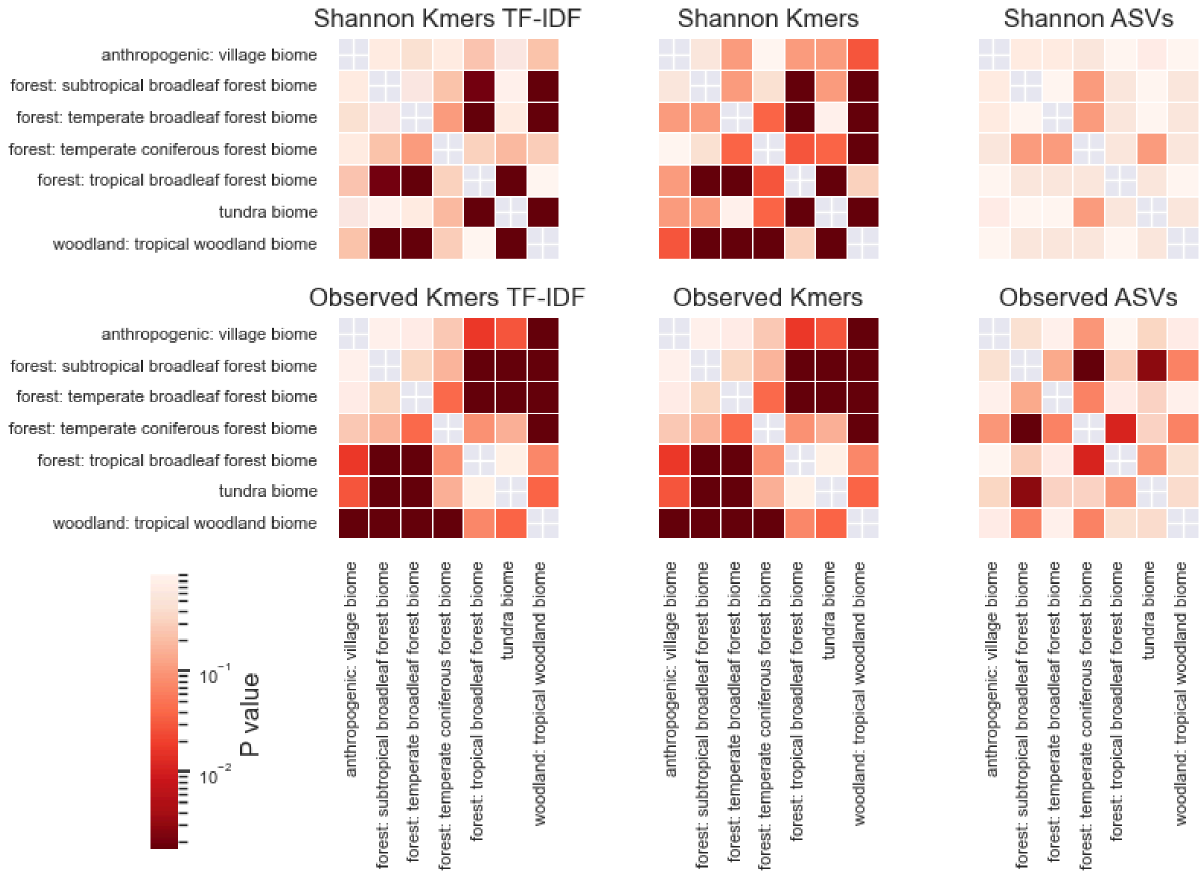

Figure S7. K-mer-based alpha diversity metrics lead to more significant differences between biomes in the Global Soil Mycobiome dataset. Heatmaps depict the False-discovery-rate-corrected P-values for pairwise ANOVA tests between each biome. K-mer size = 16.

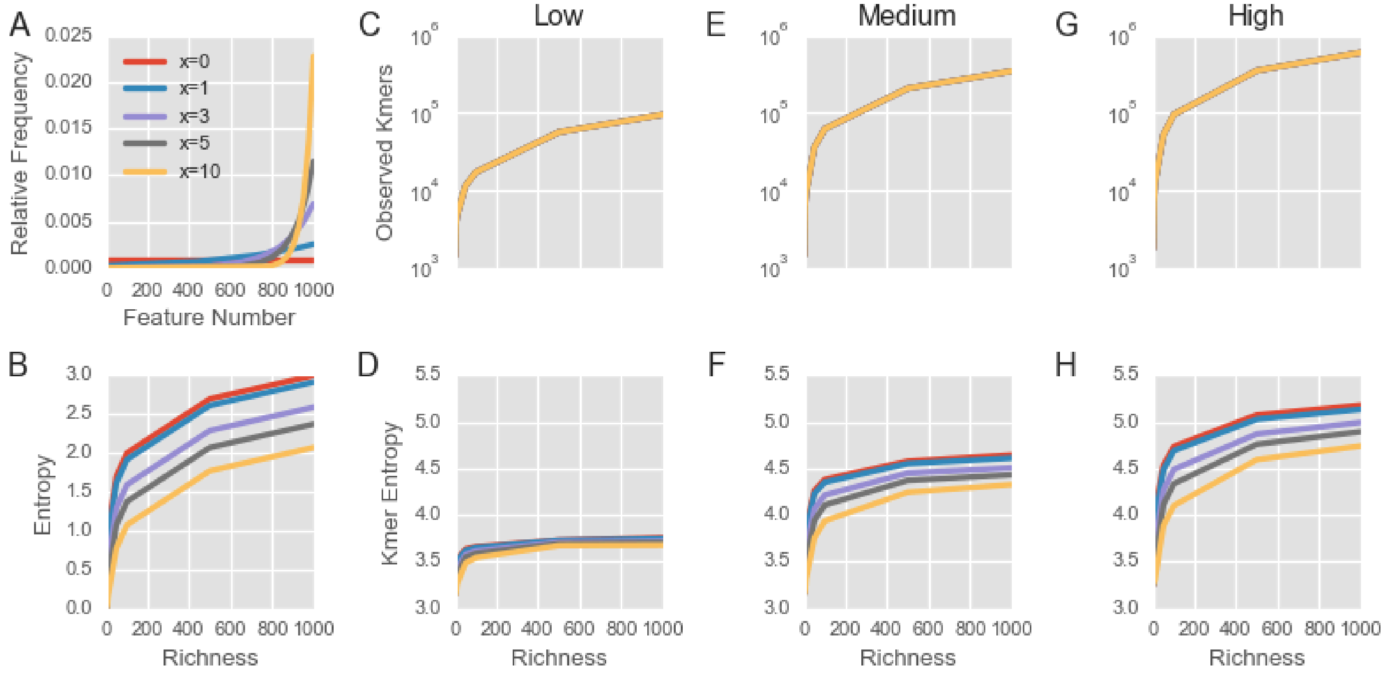

Figure S8. K-mer counting from synthetic data (k=16). A, simulated feature counts distributions following an exponential function with varying degrees of evenness. B, Shannon entropy as a function of feature (ASV) richness and evenness. C-H, observed k-mers (top) and k-mer entropy (bottom) calculated from these simulated samples in communities of low (C-D), medium (E-F), and high genetic diversity (G-H).

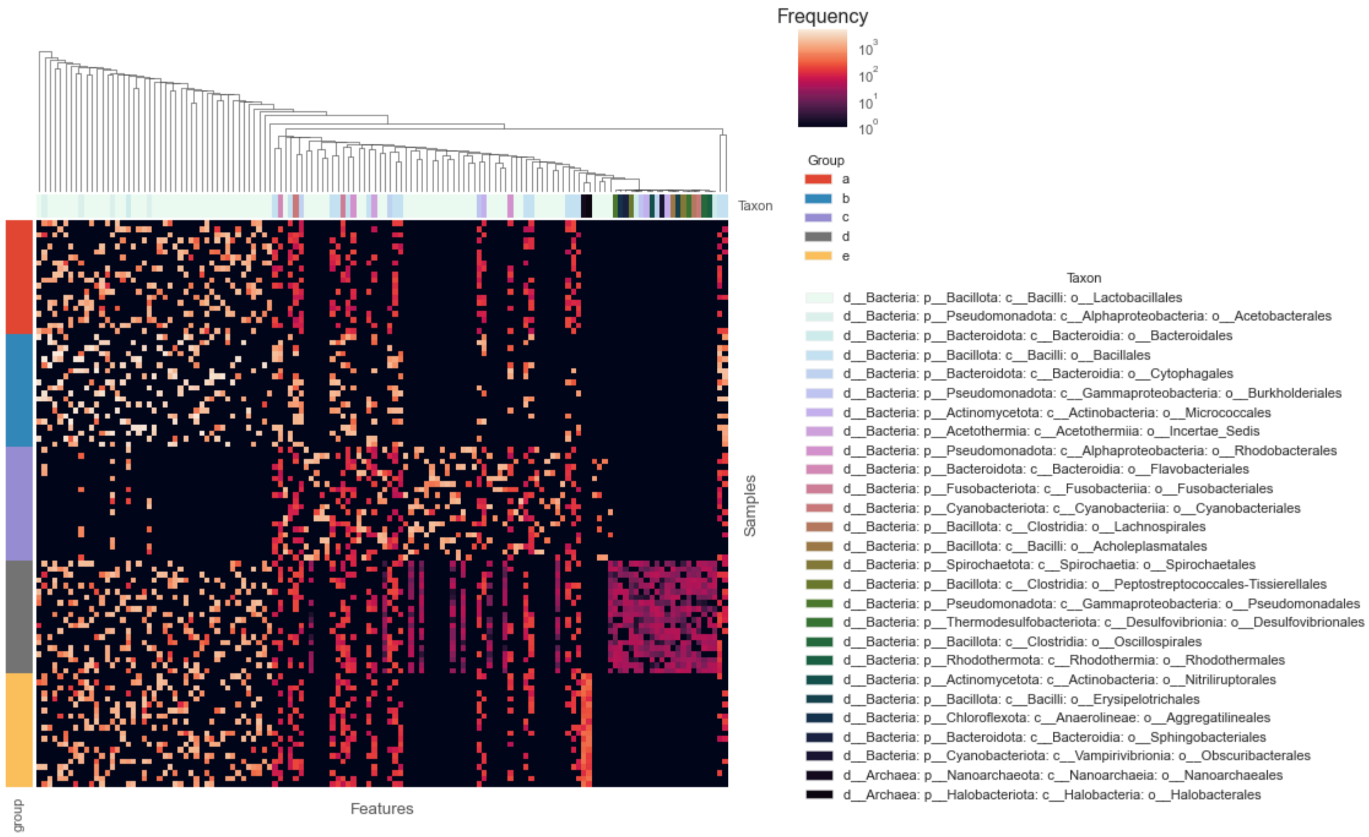

Figure S9. Composition of simulated samples used for beta diversity estimation. The clustermap shows the $\log_{10}$ frequency of each feature (columns; colored by taxonomic order and hierarchically-clustered according to co-occurrence, using default parameters of seaborn.clustermap) in each simulated sample (rows). Row margins indicate the community type of each sample; see the methods description for the description of each community type. Groups A-E correspond to the community type descriptions given in the supplemental text.

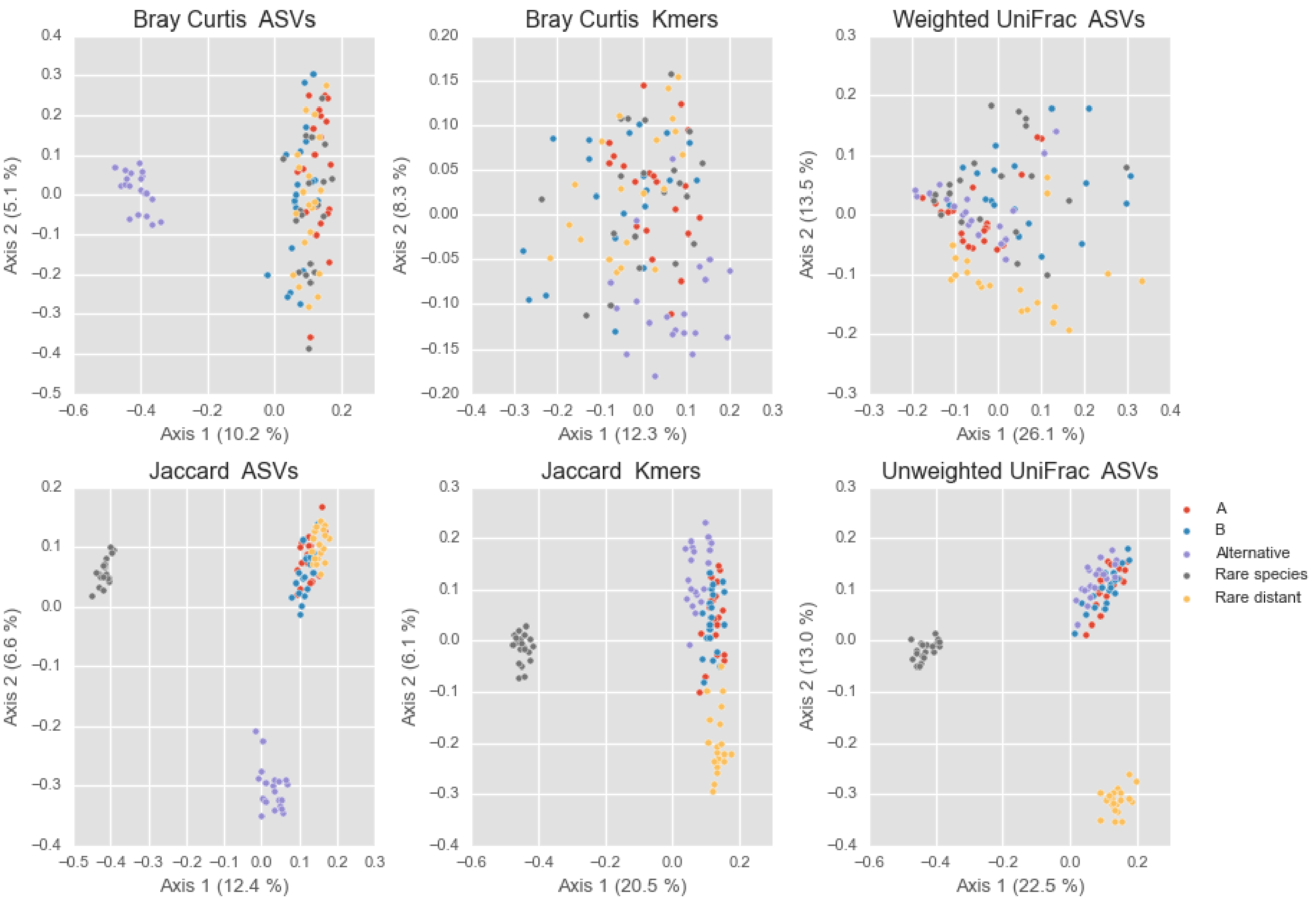

Figure S10. Beta diversity estimates from synthetic communities demonstrate strengths and weaknesses of genetically informed vs. conventional metrics. Principal coordinate plots comparing five synthetic communities with different characteristics, based on conventional (left column), k-mer based (middle column), and phylogeny-aware metrics (right column) and weighted (top row) and unweighted metrics (bottom row). To compare vs. Tables S8-S9: “Alternative” = Group C; “Rare species” = Group D; “Rare distant” = Group E. K-mer size = 16.
